# Optimal ^18^F-FDG PET/CT radiomics model development for predicting EGFR mutation status and prognosis in lung adenocarcinoma: a multicentric study

**DOI:** 10.3389/fonc.2023.1173355

**Published:** 2023-05-08

**Authors:** Yan Zuo, Qiufang Liu, Nan Li, Panli Li, Jianping Zhang, Shaoli Song

**Affiliations:** ^1^ Department of Nuclear Medicine, Fudan University Shanghai Cancer Center, Shanghai, China; ^2^ Shanghai Key Laboratory of Bioactive Small Molecules, Fudan University, Shanghai, China

**Keywords:** lung adenocarcinoma, PET/CT, epidermal growth factor receptor, radiomics, machine learning

## Abstract

**Purpose:**

To develop and interpret optimal predictive models to identify epidermal growth factor receptor (EGFR) mutation status and subtypes in patients with lung adenocarcinoma based on multicentric ^18^F-FDG PET/CT data, and further construct a prognostic model to predict their clinical outcome.

**Methods:**

The ^18^F-FDG PET/CT imaging and clinical characters of 767 patients with lung adenocarcinoma from 4 cohorts were collected. Seventy-six radiomics candidates using cross-combination method to identity EGFR mutation status and subtypes were built. Further, Shapley additive explanations and local interpretable model-agnostic explanations were used for optimal models’ interpretation. Moreover, in order to predict the overall survival, a multivariate Cox proportional hazard model based on handcrafted radiomics features and clinical characteristics was constructed. The predictive performance and clinical net benefit of the models were evaluated *via* area under receiver operating characteristic (AUC), C-index and decision curve analysis.

**Results:**

Among the 76 radiomics candidates, light gradient boosting machine classifier (LGBM) combined with recursive feature elimination wrapped LGBM feature selection method achieved best performance in predicting EGFR mutation status (AUC reached 0.80, 0.61, 0.71 in the internal test cohort and two external test cohorts, respectively). And extreme gradient boosting classifier combined with support vector machine feature selection method achieved best performance in predicting EGFR subtypes (AUC reached 0.76, 0.63, 0.61 in the internal test cohort and two external test cohorts, respectively). The C-index of the Cox proportional hazard model achieved 0.863.

**Conclusions:**

The integration of cross-combination method and the external validation from multi-center data achieved a good prediction and generalization performance in predicting EGFR mutation status and its subtypes. The combination of handcrafted radiomics features and clinical factors achieved good performance in predicting prognosis. With the urgent needs of multicentric ^18^F-FDG PET/CT trails, robust and explainable radiomics models have great potential in decision making and prognosis prediction of lung adenocarcinoma.

## Introduction

Lung adenocarcinoma is the main histological subtype of non-small cell lung cancer (1). EGFR is one of the most common tumor driver gene of lung adenocarcinoma. EGFR tyrosine kinase inhibitors (TKIs) are the main target drugs for the treatment of patients with EGFR mutations, which could improve their live condition and prolong their median survival (2, 3). Exon 19 deletion (E19) and exon 21 L858R missense (E21) are the two most common EGFR-TKIs-sensitive EGFR mutation subtypes. There is a difference in treatment strategy, response and prognosis between patients with EGFR E19 and E21 (4, 5). Therefore, identifying EGFR mutation status and subtypes are important for the treatment of patients with lung adenocarcinoma. Tissue sequence testing with biopsied or surgical tissues is the gold standard for EGFR gene mutation examination. However, many patients cannot undergo these invasive examinations due to some subjective psychological or objective physiological reasons. Furthermore, the biopsied or surgical section tissue cannot fully reflect the spatial heterogeneity and temporal heterogeneity of the tumor. Liquid biopsy is non-invasive and convenient but its stability and false-negative rate are not satisfying (6). Therefore, there is an urgent need for a non-invasive and accurate method to determine EGFR mutation status and subtypes, so as to facilitate accurate screening of patients suitable for EGFR TKIs targeted therapy and make better treatment decisions in clinical practice.

^18^Fluorine-fluorodeoxyglucose positron emission tomography-computed tomography (^18^F-FDG PET/CT) can simultaneously acquire anatomical structure and metabolic information of the tumor. There is a correlation between metabolic phenotype and gene mutations (7). It is well established that there is increased uptake of ^18^F-FDG in EGFR-mutated lung tumors (8–11). Therefore, ^18^F-FDG PET/CT-based method is promising to predict EGFR mutation status. Also, our previous study (12) has shown that machine learning model based on PET/CT handcrafted radiomics features (HRFs) achieved a good prediction performance in the identification of EGFR mutation status and subtypes in lung adenocarcinoma. Recently, the value of PET/CT-based HRFs in predicting EGFR mutation status, EGFR subtypes and prognosis had been well demonstrated (12–18), which involved different feature selection methods and machine learning algorithms. These different radiomics pipelines may lead to various predictive performance (19). Feature selection methods also have a great influence on clinical predictive models (20). However, it’s difficult to compare their performance and there is hardly no model that works well for multi-center data. To address this limitation, cross-combination of feature selection methods and classifiers based on ^18^F-FDG PET/CT HRFs have recently appeared and have proven effective in improving classification and diagnostic performance (21, 22). However, as far as we know, whether this method can be applied to predict EGFR mutation status and prognosis based on multi-center ^18^F-FDG PET/CT in lung adenocarcinoma is not fully investigated.

Furthermore, the widespread clinical application of prediction models based on machine learning is hindered because of unsatisfactory clinical trustworthiness, regardless of their huge potential for precision medicine. Interpretability is essential and extremely important to effectively understand, manage, and trust powerful artificial intelligence applications. Hence, explainable artificial intelligence (XAI) emerged and performed well in the models’ interpretation and transparency of the “black-box” problem (23). As two novel approaches of XAI, Shapley additive explanations (SHAP) and local interpretable model-agnostic explanations (LIME) have been successfully applied to medical field (24–27). However, hardly no EGFR-related predictive models involved model visualization and interpretation. Furthermore, the existing research results based on single center or small samples may have limited generalization performance, due to the differences in demographic distribution, medical imaging equipment and related imaging protocols in separate studies. Herein, we aimed to develop and interpret robust optimal predictive models to identify EGFR mutation status and subtypes based on multi-center ^18^F-FDG PET/CT data, and further construct a prognostic model to predict clinical outcome in patients with lung adenocarcinoma.

## Materials and methods

### Data collection

**Figure 1:**
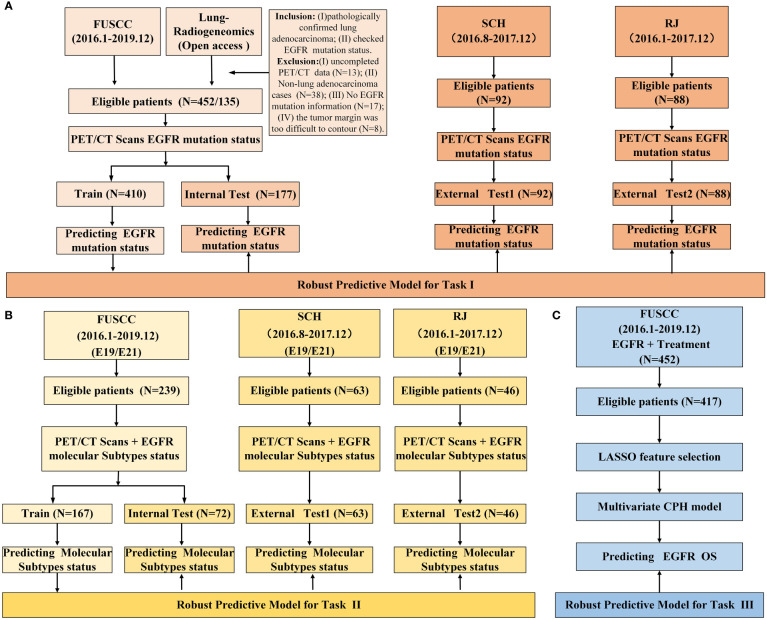
Study design and data information. **(A)** study design of predicting EGFR mutation status; **(B)** study design of predicting EGFR mutation subtypes; **(C)** study design of survival analysis.

We retrospectively reviewed 92 cases of Shanghai Chest Hospital (SCH) and 88 cases from Renji Hospital Shanghai Jiao Tong University School of Medicine (RJ) between August 2016 and July 2017, and 452 cases from Fudan University Shanghai Cancer Center (FUSCC) between January 2016 and December 2019, who were pathologically diagnosed with lung adenocarcinoma and their EGFR mutation status and subtypes were determined. Moreover, 135 cases were enrolled from the public data set (https://wiki.cancerimagingarchive.net/display/Public/NSCLC+Radiogenomics). The patients’ inclusion and exclusion criteria were summarized in **Figure 1** and **Supplementary Material** (Appendix E1). The demographic and clinical variables of the enrolled patients were detailed in **Tables 1**, **2**. The institutional review board of FUSCC approved this retrospective study and the acquirement of informed consent was waived.

**Table 1:** Demographics and clinical variables of enrolled patients in task I (N = 767).

Characters	Train group			Internal Test			External Test 1			External Test 2		
	EGFR (-)	EGFR (+)	*p*	EGFR (-)	EGFR (+)	*p*	EGFR (-)	EGFR (+)	*p*	EGFR (-)	EGFR (+)	*p*
**Number**	172	238		77	100		42	46		26	66	
**Gender**			<0.001			<0.001			<0.001			0.022
Female	59 (34.3%)	137 (57.6%)		21 (27.3%)	64 (64.0%)		8 (19.0%)	32 (69.6%)		9 (34.6%)	42 (63.6%)	
Male	113 (65.7%)	101 (42.4%)		56 (72.7%)	36 (36.0%)		34 (81.0%)	14 (30.4%)		17 (65.4%)	24 (36.4%)	
**Age**	64.0 [56.0;69.2]	62.0 [53.0;68.0]	0.018	62.6 (11.8)	61.1 (9.80)	0.365	63.9 (9.47)	63.4 (10.7)	0.819	59.7 (12.2)	57.3 (9.62)	0.367
**Location**			0.790			0.512			0.275			0.500
L-Lower	21 (12.2%)	33 (13.9%)		13 (16.9%)	10 (10.0%)		10 (23.8%)	9 (19.6%)		5 (19.2%)	5 (7.58%)	
L-Upper	50 (29.1%)	65 (27.3%)		19 (24.7%)	29 (29.0%)		5 (11.9%)	10 (21.7%)		7 (26.9%)	24 (36.4%)	
R-Lower	28 (16.3%)	42 (17.6%)		12 (15.6%)	18 (18.0%)		12 (28.6%)	7 (15.2%)		6 (23.1%)	12 (18.2%)	
R-Middle	16 (9.30%)	15 (6.30%)		6 (7.79%)	4 (4.00%)		4 (9.52%)	2 (4.35%)		3 (11.5%)	8 (12.1%)	
R-Upper	57 (33.1%)	83 (34.9%)		27 (35.1%)	39 (39.0%)		11 (26.2%)	18 (39.1%)		5 (19.2%)	17 (25.8%)	
**TNM**			0.011			0.003			0.921			0.760
I	67 (39.0%)	120 (50.4%)		25 (32.5%)	56 (56.0%)		7 (16.7%)	10 (21.7%)		9 (34.6%)	29 (43.9%)	
II	23 (13.4%)	28 (11.8%)		7 (9.09%)	15 (15.0%)		10 (23.8%)	9 (19.6%)		2 (7.69%)	7 (10.6%)	
III	35 (20.3%)	56 (23.5%)		22 (28.6%)	14 (14.0%)		17 (40.5%)	18 (39.1%)		6 (23.1%)	14 (21.2%)	
IV	30 (17.4%)	25 (10.5%)		19 (24.7%)	11 (11.0%)		8 (19.0%)	9 (19.6%)		9 (34.6%)	16 (24.2%)	
Others	17 (9.88%)	9 (3.78%)		4 (5.19%)	4 (4.00%)		0	0		0	0	
**Grades**			0.001			0.001			0.101			0.128
G1	14 (8.14%)	7 (2.94%)		8 (10.4%)	3 (3.00%)		1 (2.38%)	3 (6.52%)		3 (11.5%)	11 (16.7%)	
G2	54 (31.4%)	84 (35.3%)		15 (19.5%)	39 (39.0%)		18 (42.9%)	28 (60.9%)		9 (34.6%)	35 (53.0%)	
G3	52 (30.2%)	103 (43.3%)		25 (32.5%)	41 (41.0%)		23 (54.8%)	15 (32.6%)		14 (53.8%)	20 (30.3%)	
Others	52 (30.2%)	44 (18.5%)		29 (37.7%)	17 (17.0%)		0	0		0	0	
**LD**	2.40 [1.40;3.30]	2.50 [1.70;3.50]	0.130	2.50 [1.50;3.80]	2.40 [1.50;3.00]	0.402	3.30 [2.50;4.72]	2.90 [2.00;4.15]	0.174	3.30 [2.50;4.50]	2.59 [1.70;3.92]	0.051
**SUVmax**	7.95 [4.50;11.2]	5.90 [3.50;10.1]	0.090	8.60 [5.00;11.5]	6.10 [2.92;9.10]	0.004	12.1 [8.15;14.8]	10.2 [5.82;14.6]	0.169	11.4 [8.53;14.8]	7.50 [4.40;12.0]	0.003

Annotations: L, left; R, right; LD, longest diameters; G, grade; EGFR, epidermal growth factor receptor; +, mutation mutant-type; -, mutation wild-type.

**Table 2:** Demographics and clinical variables of enrolled patients in task II (N = 348).

Characters	Train group			Internal Test			External Test 1			External Test 2		
	E19	E21	*p*	E19	E21	*p*	E19	E21	*p*	E19	E21	*p*
**Number**	70	97		37	35		27	19		30	33	
**Gender**			0.564			0.786			0.854			0.578
Female	46 (65.7%)	63 (64.9%)		20 (54.1%)	21 (60.0%)		18 (66.7%)	14 (73.7%)		17 (56.7%)	22 (66.7%)	
Male	24 (34.3%)	34 (35.1%)		17 (45.9%)	14 (40.0%)		9 (33.3%)	5 (26.3%)		13 (43.3%)	11 (33.3%)	
**Age**	58.0 (9.19)	60.1 (9.84)	0.159	60.0 (10.4)	62.2 (8.33)	0.312	61.0 (12.0)	66.9 (7.39)	0.042	55.4 (8.11)	59.2 (10.9)	0.112
**Location**			0.007			0.960			0.051			0.751
L-Lower	11 (15.7%)	9 (9.28%)		6 (16.2%)	7 (20.0%)		7 (25.9%)	2 (10.5%)		2 (6.67%)	3 (9.09%)	
L-Upper	11 (15.7%)	29 (29.9%)		11 (29.7%)	10 (28.6%)		2 (7.41%)	8 (42.1%)		9 (30.0%)	14 (42.4%)	
R-Lower	21 (30.0%)	11 (11.3%)		8 (21.6%)	5 (14.3%)		5 (18.5%)	2 (10.5%)		6 (20.0%)	4 (12.1%)	
R-Middle	2 (2.86%)	7 (7.22%)		3 (8.11%)	3 (8.57%)		2 (7.41%)	0 (0.00%)		5 (16.7%)	3 (9.09%)	
R-Upper	5 (35.7%)	41 (42.3%)		9 (24.3%)	10 (28.6%)		11 (40.7%)	7 (36.8%)		8 (26.7%)	9 (27.3%)	
**TNM**			0.030			0.106			0.332			0.847
I	29 (41.4%)	51 (52.6%)		19 (51.4%)	21 (60.0%)		8 (29.6%)	2 (10.5%)		12 (40.0%)	16 (48.5%)	
II	12 (17.1%)	10 (10.3%)		3 (8.11%)	8 (22.9%)		6 (22.2%)	3 (15.8%)		3 (10.0%)	4 (12.1%)	
III	16 (22.9%)	30 (30.9%)		9 (24.3%)	3 (8.57%)		9 (33.3%)	9 (47.4%)		7 (23.3%)	7 (21.2%)	
IV	13 (18.6%)	6 (6.19%)		6 (16.2%)	3 (8.57%)		4 (14.8%)	5 (26.3%)		8 (26.7%)	6 (18.2%)	
**Grades**			0.613			0.295			0.790			0.782
G1	0 (0.00%)	1 (1.03%)		0 (0.00%)	1 (2.86%)		2 (7.41%)	1 (5.26%)		4 (13.3%)	6 (18.2%)	
G2	24 (34.3%)	38 (39.2%)		13 (35.1%)	16 (45.7%)		15 (55.6%)	13 (68.4%)		16 (53.3%)	19 (57.6%)	
G3	34 (48.6%)	47 (48.5%)		13 (35.1%)	13 (37.1%)		10 (37.0%)	5 (26.3%)		10 (33.3%)	8 (24.2%)	
Others	12 (17.1%)	11 (11.3%)		11 (29.7%)	5 (14.3%)		0	0		0	0	
**LD**	2.50 [1.70;3.50]	2.50 [2.00;3.50]	0.744	2.20 [1.50;3.00]	2.50 [1.80;3.35]	0.390	2.80 [1.95;4.10]	3.10 [2.05;4.25]	0.671	2.60 (1.34)	2.94 (1.43)	0.351
**SUVmax**	5.60 [4.18;8.80]	6.40 [3.30;10.3]	0.991	4.60 [2.85;8.50]	5.50 [3.28;9.25]	0.642	9.92 (5.13)	11.4 (6.80)	0.432	6.70 [3.80;9.00]	7.69 [4.60;12.0]	0.516

Annotations: L, left; R, right; LD, longest diameters; G, grade; E19, Exon 19 deletion of epidermal growth factor receptor; E21, exon 21 L858R missense of epidermal growth factor receptor.

### EGFR mutation status detection

In this study, EGFR mutation status of 3 hospitals obtained by next-generation sequencing test (Illumina high-throughput sequencing, 68-gene panel detection general kit of Burning Rock Medical laboratory, Guangzhou) or an amplification refractory mutation system real-time technology with ARMS (AmoyDx EGFR Mutations Detection Kit). And the EGFR mutation status were retrospectively collected from the medical record system of the hospitals.

### Image acquisition and preprocessing

Herein, scanners of three hospitals all belong to Siemens Medical Systems, Erlangen, Germany, that are Biograph mCT, Biogragh 16HR and Biogragh mCT-s. The details of the scanner information and imaging protocols were listed in Supplementary material (**Table S1** and Appendix E2). The PET image datasets were reconstructed using CT data for attenuation correction. Then, PET and CT images were preprocessed according to guidelines (28), including resampling all images to a voxel size of 1×1×1mm^3^ using B-spline interpolation, and both PET and CT images were subjected to intensity discretization.

### Tumor segmentation and HRFs extraction

ITK-SNAP version 3.8.0-beta (https://www.itksnap.org/), an open-source image analytic platform, was used to segment the primary tumor regions. Volumes of interest (VOIs) of primary tumors for CT were drawn on the lung window (window width: 1600HU; window level: -600HU). The robustness and repeatability of HRFs were evaluated. Both PET and CT HRFs of the VOIs were automatically calculated using Python 3.7 and the Pyradiomics package. The formulas and matrices for texture feature calculation followed the image biomarker standardization initiative (29). Further, 2,380 HRFs were extracted from the PET and CT images, respectively. The detailed tumor segmentation and HRFs extraction were available in **Supplementary Material** (Appendix E3, **Table S2** and **Table S3**).

### Feature selection

HRFs selection and predictive models’ development were separately performed on train groups of task I (EGFR mutated type *vs.* EGFR wild type) and task II (EGFR E19 *vs.* EGFR E21). In our work, to reduce the feature variability caused by different PET/CT scanners and associated protocols, all HRFs were normalized using z scores and harmonized using ComBat (30, 31). The Combat function of non-parametric version from R package (R statistical software version 4.1.2) were used. Both 2 tasks would do a feature cleaning preprocess to remove constant or quasi-constant features, and duplicated features. Firstly, the missing value was filled with conditional mean completer. Then, removed features with a single unique value and low variance, and further removed collinear features as identified by a correlation coefficient greater than 0.75. Finally, mutual information was used to further removed redundant features.

As for task I and task II, 19 feature selection methods, which belong to wrapper and embedded categories, were used for feature selection. Sequential forward selection (SFS), sequential backward selection (SBS) and recursive feature elimination (RFE) wrapped 4 classifiers, respectively, that were light gradient boosting machine (LGBM), extreme gradient boosting (XGB), random forest (RF) and logistic regression (LR), and then 12 wrapper feature selection candidates were obtained. Seven embedded feature selection methods were enrolled, including LGBM, XGB, RF, LR, K-nearest Neighbor (KNN), support vector machine (SVM) and least absolute shrinkage and selection operator (LASSO). In our study, embedded feature selection methods ranked features by calculating the AUC based on the individual feature.

As for task III, LASSO Cox regression model was used to select the most useful prognostic HRFs in the entire dataset. Univariate and multivariate analyses with CPH regression determined the clinical risk factors of overall survival (OS).

### Development and evaluation of predictive models

**Figure 2:**
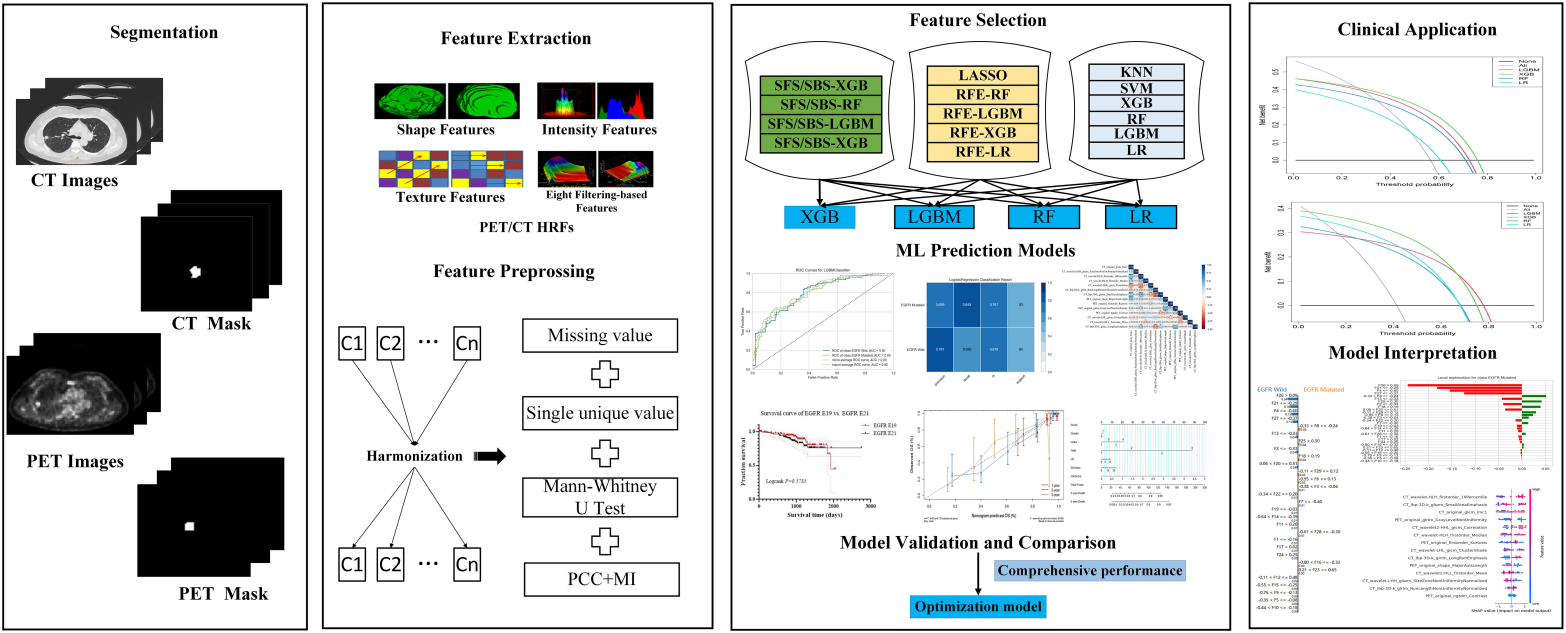
Workflow.

LGBM, XGB, RF and LR were used to develop predictive models. We used a grid search technique with 10-fold cross-validation to determine the optimal parameter values of various kernel function. In task І, the model was trained on a train group (n=410) and tested in an internal test group (n=177). And in task II, E19 and E21 mutation subtypes from EGFR mutated group were considered as negative and positive labels, respectively. The E19/E21 mutation subtypes prediction model was trained on a train group (n=167) and tested in an internal test group (n=72). In both task І and II, data from SCH and RJ were separately used for external test. The study design and workflow were detailed in **Figures 1**, **2**, respectively.

Confusion matrix, classification report, feature importance and ROC curve, which programmed and calculated with the Python Scikit-learn and Yellowbrick package (Python version 3.7, Yellowbrick version 1.5, https://www.scikit-yb.org/, Scikit-learn version 1.0.2, http://scikit-learn.org/), as well as the area under the curve (AUC), precision, recall, F1-score, and accuracy (ACC), were used to evaluate the performance of the model. Decision curve analysis (DCA) was used to evaluate the clinical applicability of the predictive models. Optimal models for task І and task II were determined from 76 predictive models, according to the comprehensive AUC performance of one internal test and two external test groups, respectively.

As for task III, a radiomics score (PET/CT-RadScore) was calculated for each patient through a linear combination of the selected features weighted according to their respective coefficient. The clinical risk factors combined with PET/CT-RadScore were then used to build nomogram model. The Harrell’s concordance index (C-index) was calculated to evaluate the performance of nomogram model. Calibration curve was used to evaluate the goodness-of-fit of the nomogram model. The prognosis of EGFR-TKIs targeted therapy and chemotherapy in lung adenocarcinoma patients with different EGFR mutation status and subtypes was compared.

Radiomics quality score (RQS) and the transparent reporting of a multivariable prediction model for individual prognosis or diagnosis (TRIPOD) systems were used to evaluate the quality of this radiomics research.

### Interpretation of optimal predictive models

In order to improve the interpretability of the models, feature importance, and SHAP and LIME were used for optimal models’ global and local interpretation in task І and II. The detailed description of them could be found at **Supplementary Material** (Appendix E4).

### Statistical analysis

Statistical analyses were performed using the GraphPad Prism version 6. The Shapiro-Wilks test was used to determine whether clinical data fit a normal distribution. The difference and statistical analysis in the related clinical information in the training and test cohorts were assessed using descrTable function of compareGroups R package. Discrete variables were expressed as medians and ranges. The Kaplan-Meier method was used and compared by a two-sided log-rank tests. p < 0.05 was considered statistically significant.

## Results

### Demographic and clinical information

We enrolled 767 patients from 4 cohorts who were pathologically diagnosed with lung adenocarcinoma and EGFR mutation status. In train group of task I, there is a significant difference in gender, age, TNM staging, and grades between EGFR mutant and EGFR wild groups (p < 0.001, 0.018, 0.011, 0.001, respectively), and in train group of task II, there is a significant difference in primary tumor location (p = 0.007) and TNM staging (p = 0.030). Gender, TNM staging, grades, primary tumor’s longest diameter (LD) and SUVmax were treated as clinical risk factors in task III. The clinical information of the enrolled patients was detailed in **Table 1** and **Table 2**.

### Feature reduction and selection

**Figure 3:**
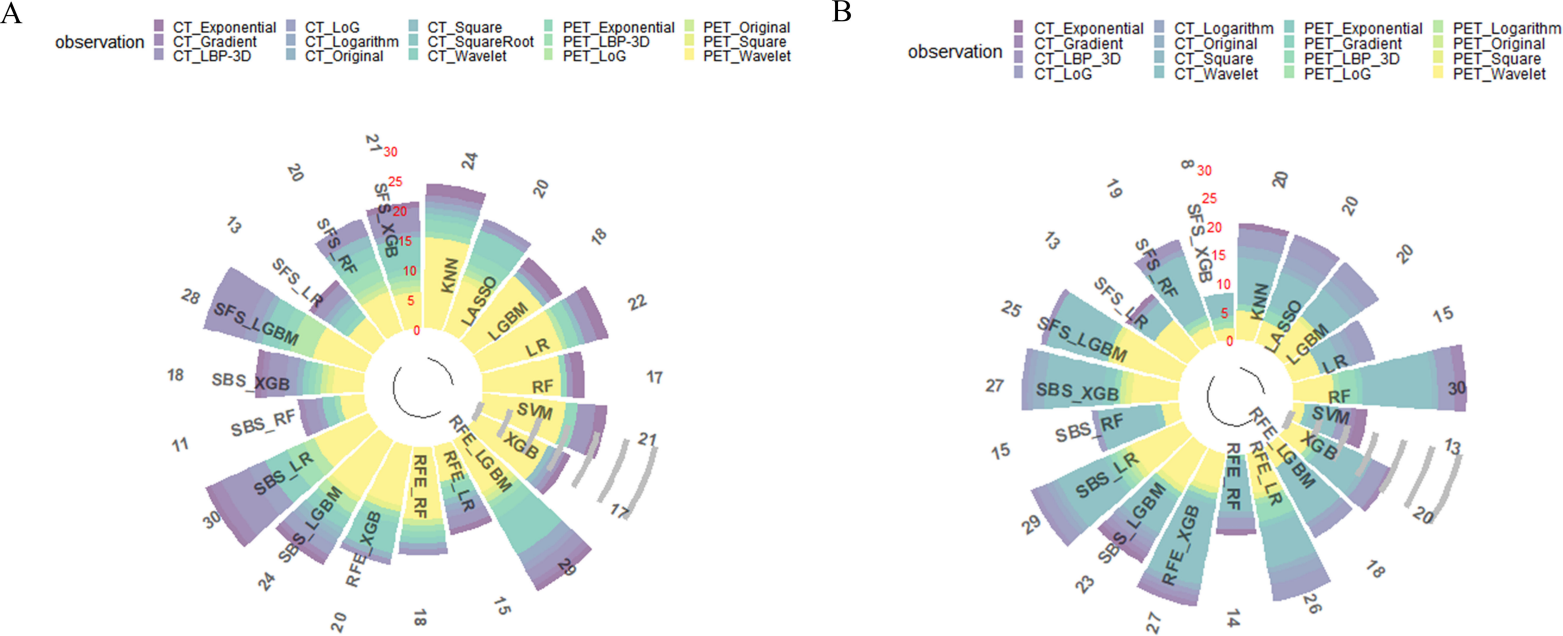
Results of all feature selection methods. **(A)** feature selection result of predicting EGFR mutation status; **(B)** feature selection result of predicting EGFR mutation subtypes. LBP, local binary pattern. LoG, Laplacian of Gaussian.

The intra –and interclass correlation coefficients values of the HRFs extracted from the different radiologists and the different scanners were all greater than 0.75, reflecting good consistency. After feature preprocessing pipeline, 213 features (111 CT HRFs and 102 PET HRFs) remained in task I. As for task II, 288 features (142 CT HRFs and 146 PET HRFs) remained. In both task I and II, CT features from wavelet, local binary pattern (LBP) 3D and Laplacian of Gaussian (LoG) filters accounted for the largest proportion, and PET features from wavelet, LBP 3D and exponential filters accounted for the largest proportion. The correlation of enrolled PET/CT HRFs of optimal model in task I and task II were shown in **Figures S2**, **S4**, respectively. Total number and categories of HRFs of 19 feature selection methods in task I and task II were shown in **Figure 3**. The detailed AUC thresholds of feature selection methods in task I and task II were shown in **Table S4**.

### Model development and evaluation

**Figure 4:**
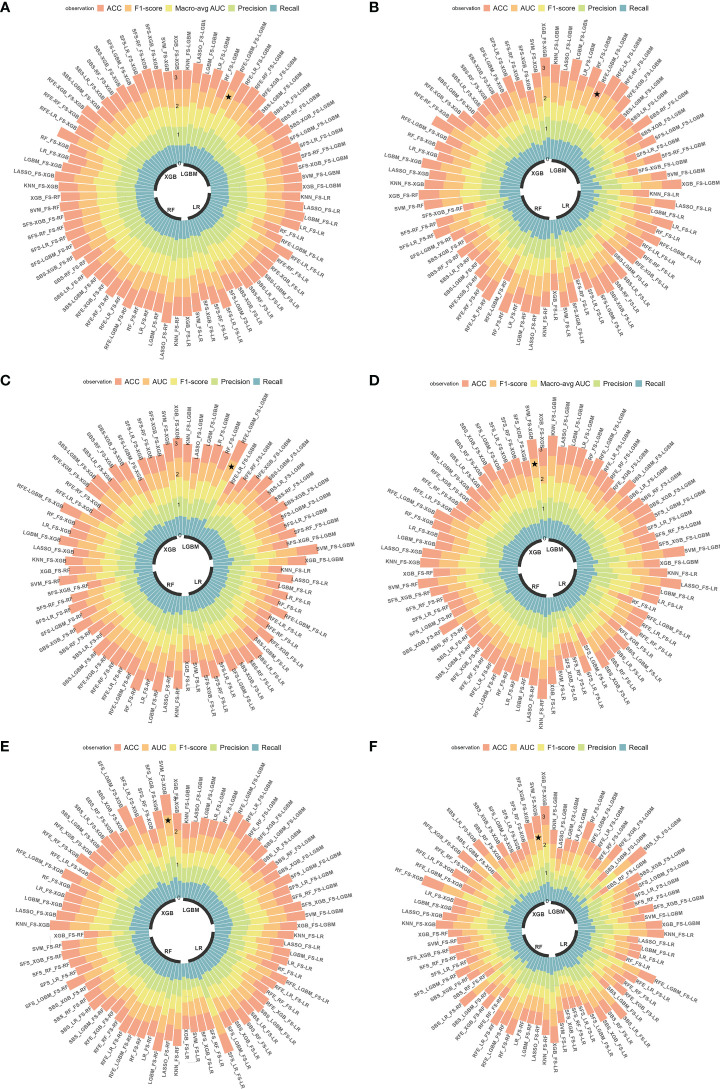
Predictive performance of all radiomics models. **(A-C)** AUC, ACC, precision, recall and F1-score in the internal test cohort and 2 external test cohorts in predicting EGFR mutation status; **(D-F)** AUC, ACC, precision, recall and F1-score in the internal test cohort and 2 external test cohorts in predicting EGFR mutation subtypes. ★, the optimal model. Macro-avg, Macro average.

**Figure 5:**
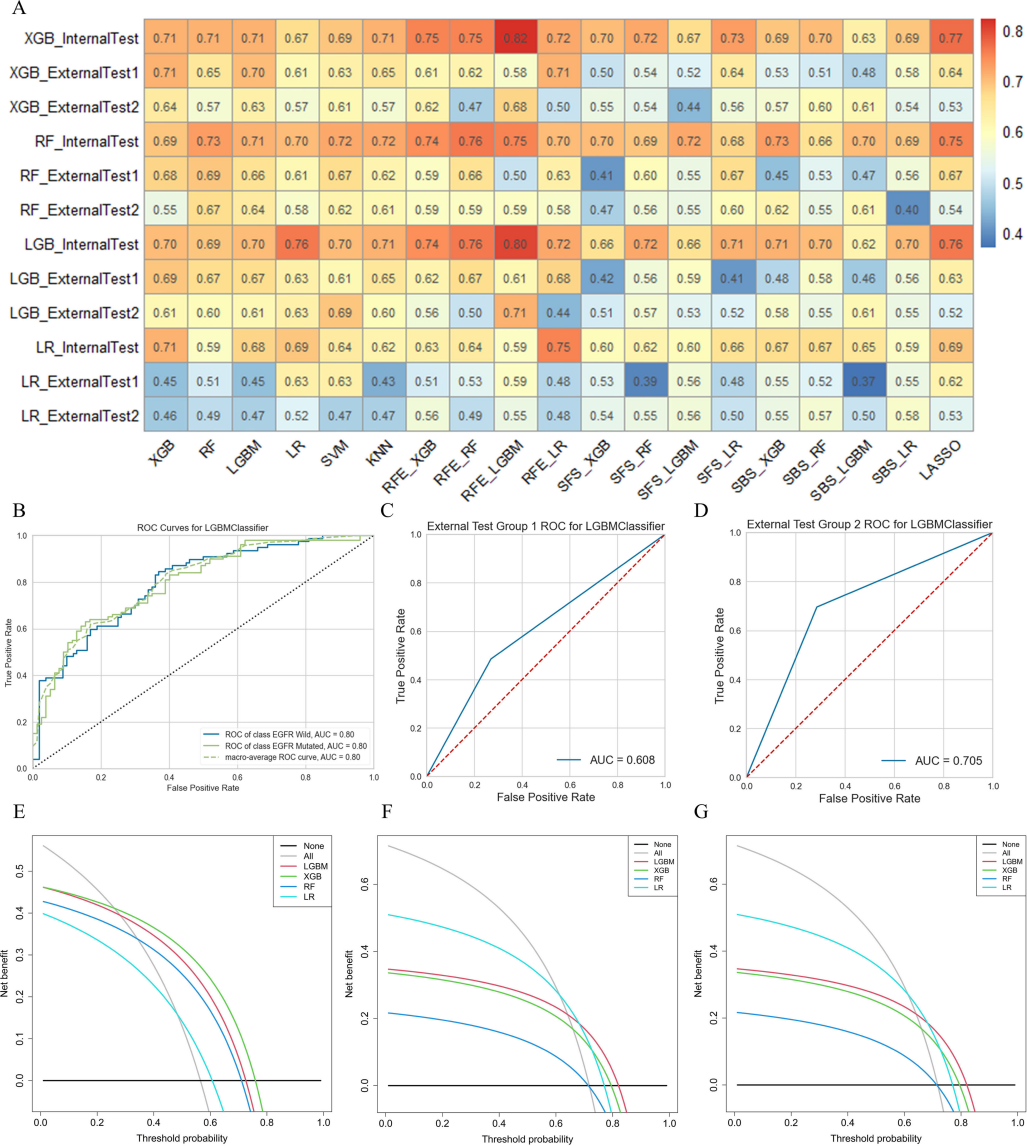
The performance of models in predicting EGFR mutation status. **(A)** AUC performance of all models based on cross-combination method. **(B-D)** AUC performance of internal test group, external test group 1 and external test group 2 of optimal predictive model. **(E-G)** Clinical net benefit of internal test group, external test group 1 and external test group 2 of optimal predictive model.

In this study, 76 predictive models were obtained using cross-combination of 19 feature selection methods and 4 classifiers (XGB, LGBM, RF and LR) for prediction. All the models performed binary predictions. The performance of all the predictive models in task I and task II was shown in **Figure 4**. As for task I, the results indicated that 29 HRFs selected from RFE - LGBM feature selection method and then LGBM classifier used for prediction achieved the best performance (AUC = 0.80, ACC = 0.72, precision = 0.72, recall = 0.71, F1-score = 0.71 in the internal test cohort; AUC = 0.61, ACC = 0.55, precision = 0.59, recall = 0.61, F1-score = 0.55 in the external test cohort 1; AUC = 0.71, ACC = 0.70, precision = 0.70, recall = 0.70, F1-score = 0.70 in the external test cohort 2) among the 76 feature selection-classification radiomics candidates (**Figures 4A–C**, **Figures 5B–D**, **Figure S1A**).The statistical results and correlation heat map of the 29 HRFs for task I could be found at **Table S5** and **Figure S2**.

**Figure 6:**
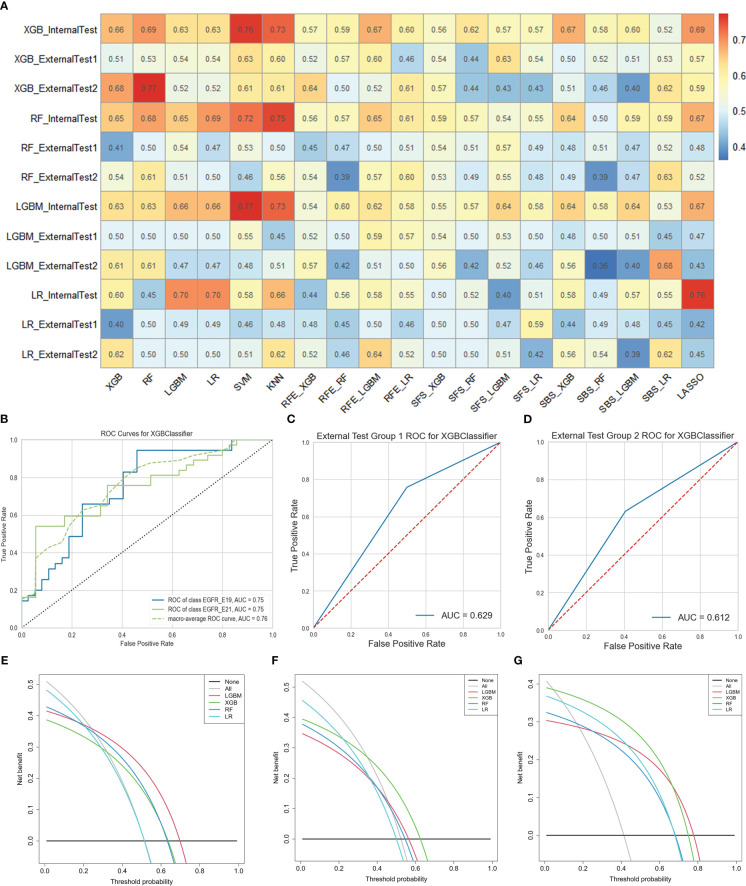
The performance of models in predicting EGFR mutation subtypes. **(A)** AUC performance of all models based on cross-combination method. **(B-D)** AUC performance of internal test group, external test group 1 and external test group 2 of optimal predictive model. **(E-G)** Clinical net benefit of internal test group, external test group 1 and external test group 2 of optimal predictive model. .

As for task II, the results indicated that 13 HRFs selected from SVM feature selection method and then XGB classifier used for prediction achieved the best performance (AUC = 0.76, ACC = 0.65, precision = 0.66, recall = 0.65, F1-score = 0.65 in the internal test cohort; AUC = 0.63, ACC = 0.63, precision = 0.64, recall = 0.63, F1-score = 0.63 in the external test cohort 1; AUC = 0.61, ACC = 0.61, precision = 0.61, recall = 0.61, F1-score = 0.61 in the external test cohort 2) among the 76 feature selection-classification radiomics candidates (**Figures 4D–F**, **Figures 6B–D**, **Figure S3A**). Precision, recall, F1-score were in the macro average version. The statistical results and correlation heatmap of the 13 HRFs for task II could be found at **Table S6** and **Figure S4**. Clinical net benefit and confusion matrix of three test groups of optimal predictive model in task I and task II were calculated (**Figures 5E–G**, **Figures S1B-D**, **Figures 6E–G**, **Figures S3B-D**). DCA curves also showed that two optimal predictive models have a certain clinical benefit. AUC heatmaps of all models based on cross-combination method in task I and task II were shown in **Figures 5A** and **6A**.

Overall, both in task I and II, RFE and embedded feature selection methods always achieved good predictive performance, which outperformed SBS and SFS feature selection methods. Our study strictly adhered to the radiomics process, and the actual RQS could reach 23 (**Table S8**). It is worth mentioning that the TRIPOD checklist (32) was shown in **Table S9**.

### Interpretation of optimal predictive models

**Figure 7:**
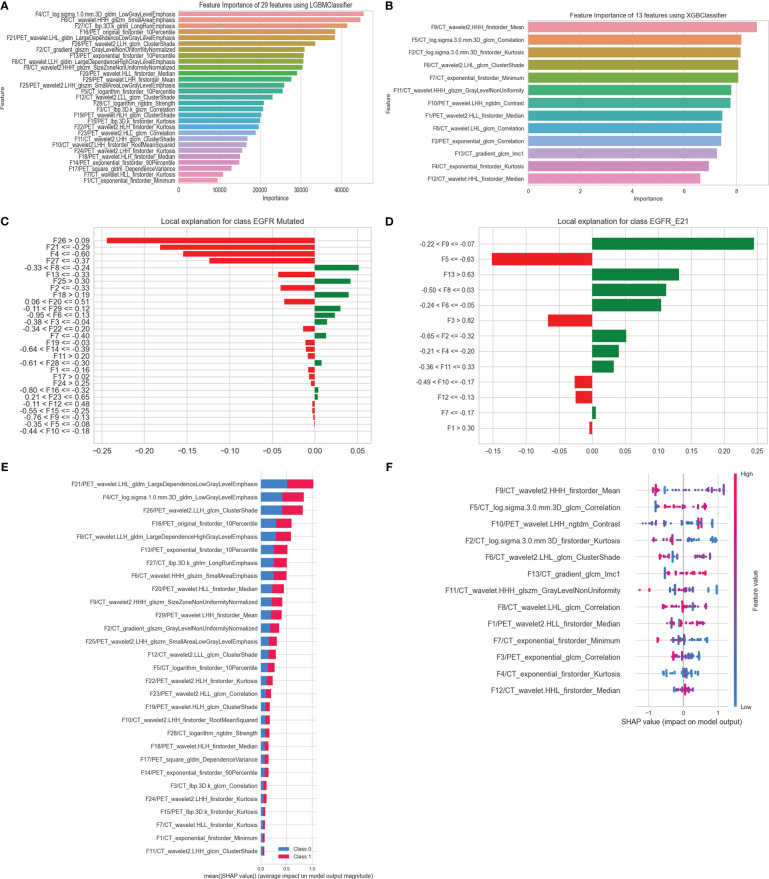
Interpretation of optimal models in predicting EGFR mutation status and subtypes. **(A)** Feature importance of 29 HRFs; **(C)** the rank of HRFs and their interactions calculated by LIME (patient index 520, who was pathologically diagnosed with an EGFR mutant-type); **(E)** Mean SHAP value of 29 HRFs, in predicting EGFR mutation status. **(B)** Feature importance of 13 HRFs; **(D)** the rank of handcrafted radiomics features and their interactions calculated by LIME (patient index 9, who was pathologically diagnosed with an E21 subtype); **(F)** SHAP value of 13 HRFs, in predicting EGFR mutation subtypes.

In this study, feature importance and SHAP were used for optimal models’ global interpretation, and optimal models’ local interpretation was analyzed using LIME. The importance of features decreased from top to bottom (**Figures 7A, B**). Feature importance value was amplified 100 times before visualization. As for task I, the mean of SHAP value bars of 29 HRFs showed the average impact in predicting EGFR mutation status and indicated the feature ranking interpretation of the LGBM model (**Figure 7E**). As for task II, the spot plots of SHAP value provided the influence of 13 HRFs on XGB model outputs (**Figure 7F**).

Interpretation of sample prediction results need random drawing of samples to make model predictions and obtained visualizations of how model predictions are performed through the LIME algorithm. In this study, both in task I and task II, the rank of HRFs was generally similar among feature importance, SHAP and LIME (**Figures 7C, D**). This is sufficient to verify the practicability of the LGBM model and XGB model and could help to increase their clinical trustworthiness in predicting EGFR mutation status and subtypes, and further to assist in providing clinical decision support.

### Survival analysis

**Figure 8:**
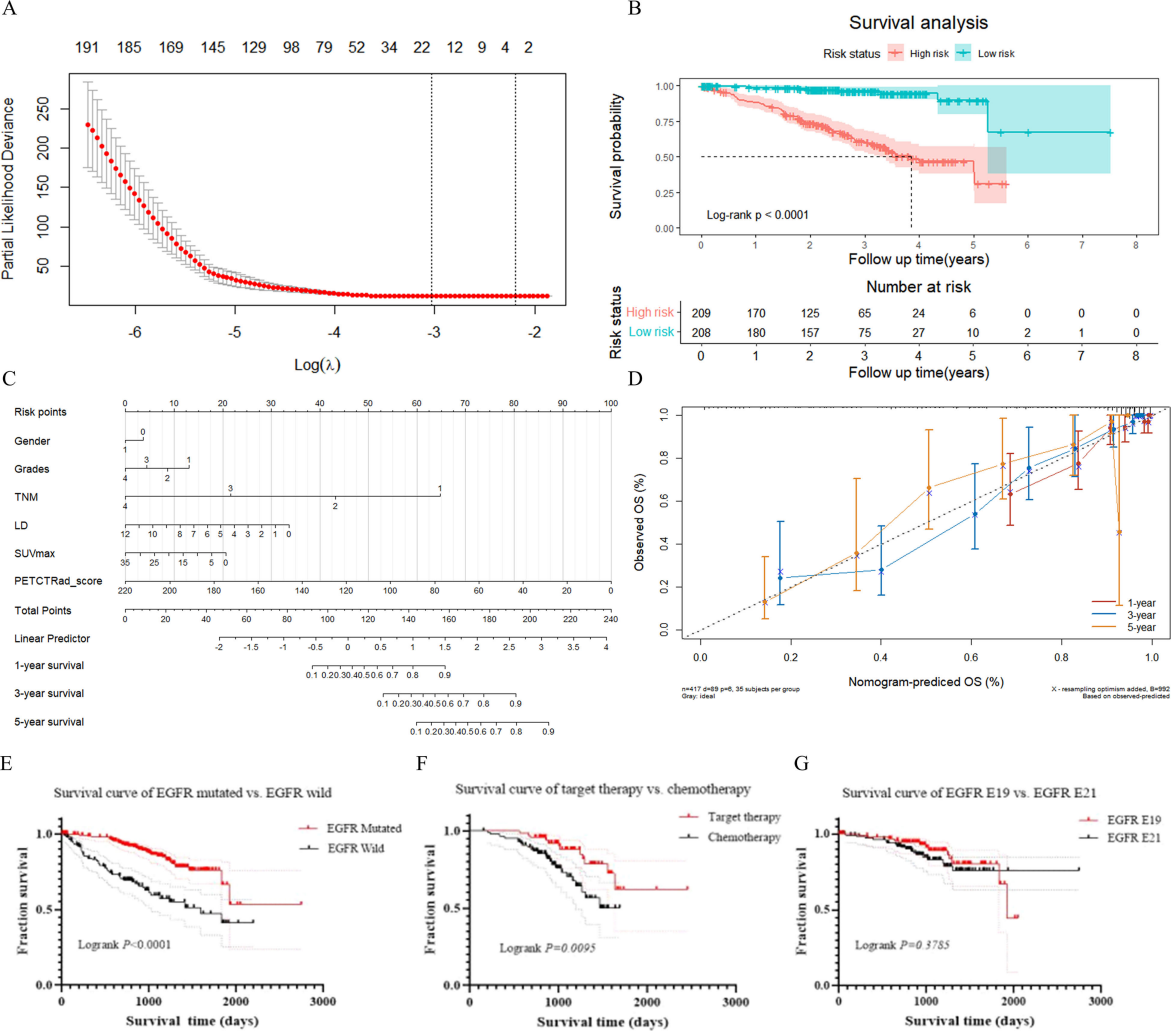
Results of survival analysis. **(A)** LASSO feature selection. **(B)** Kaplan-Meier survival analysis. **(C)** nomogram model. **(D)** calibration curve at the 1-year, 3-year, and 5-year time points. **(E)** survival curve of lung adenocarcinoma patients with EGFR mutant-type and EGFR wild-type. **(F)** survival curve of target therapy and chemotherapy in lung adenocarcinoma patients with EGFR mutant-type. **(G)** survival curve of lung adenocarcinoma patients with EGFR E19 and EGFR E21 subtypes.

Out of a total of 452 cases at FUSCC, 35 patients were lost to follow-up, 89 patients died during follow-up, and 328 patients were alive at the end of follow-up. The 1-, 3- and 5-year OS rates were 83.93%, 33.57% and 3.83%, respectively. The median survival time for the 417 patients was 29.03 months [interquartile range (IQR), 21.34-40.48 months]. Twenty-two PET/CT-based HRFs were selected as prognosis factors using LASSO regression analysis (**Figure 8A**, **Figure S5**). Further, Kaplan-Meier survival analysis and Log-rank test verified that the 22 HRFs were useful prognostic predictors (Log-rank p < 0.0001, **Figure 8B**). The independent clinical factors (gender, TNM staging, grades, LD) and PET (SUVmax) predictor that were significant in the multivariate Cox regression analysis were used to build the combined models for predicting OS (**Figure 8C**). PET/CT-RadScore was calculated for each patient through a linear combination of the 22 HRFs weighted according to their respective coefficient. PET/CT-RadScore combined with 5 clinical risk factors demonstrated a good predictive survival performance, with a C-index of 0.863. The information of the 22 HRFs for task III could be found at **Table S7** and **Figure S6**. The calibration curve for predicting the probability of OS at 1, 3, or 5 years for each model after 1000 bootstrap replicates was shown in **Figure 8D**, which showed satisfactory agreement between the estimations and actual observations.

There were 281 patients with EGFR mutant-type and 137 patients with EGFR wild-type. There was a significant difference in survival time between those patients with EGFR mutant-type and EGFR wild-type in total (p < 0.0001). Among those patients with EGFR mutant-type, 85 patients underwent targeted therapy and 137 underwent chemotherapy. The prognosis for targeted therapy was better than that for chemotherapy. There was a significant difference in survival time between targeted therapy and chemotherapy for patients with EGFR mutant-type (p = 0.0095). One hundred and three patients with E19 and 124 with E21 mutations in total. There was no significant difference in survival time between those with E19 and E21 mutations (p = 0.3785). Survival curves for above were presented in **Figures 8E–G**.

## Discussion

In this study, firstly, we successfully constructed separately two cross-combination predictive models for EGFR mutation status and subtypes. Then, optimal models were determined from 76 predictive models according to the comprehensive AUC performance of an internal test group and 2 external test groups. The predictive model using RFE - LGBM and LGBM achieved the best performance in task I, and the predictive model using SVM and XGB achieved the best performance in task II. Furthermore, we performed model visualization and interpretation. Besides, we also built a multivariate CPH model based on clinical risk factors and robust ^18^F-FDG PET/CT HRFs for OS prediction in lung adenocarcinoma. Together, our results may provide important information in predicting EGFR mutation status, EGFR subtypes and OS, to help lung adenocarcinoma treatment and prognosis.

Identifying EGFR mutation status and main subtypes of patients with lung adenocarcinoma are beneficial in making EGFR-TKIs-related treatment strategies in clinic. Sho et al. (33) used the same public data set as ours to build models predicting histological subtype and EGFR mutation status of non-small cell lung cancer on ^18^F-FDG PET/CT, and XGB classifier achieved better performance (AUC = 0.659) than gradient tree boosting, Bayesian optimization and RF classifiers. Yang et al. (17) introduced two RF-based predictive models and a multivariate CPH model for predicting EGFR mutation status, EGFR subtypes and OS in total 174 patients with lung adenocarcinoma. And their results showed that the AUC of train and validation group were 0.77 and 0.71 for EGFR mutant/wild-type prediction, and reached 0.82 and 0.73 in EGFR E19/E21 prediction. However, they did not involve cross-combination method and further multi-center data validation. Our study built two separately cross-combination models to identify EGFR mutation status and subtypes. In the internal test group and two external test groups, the optimal model in EGFR mutant/wild-type prediction could reach an AUC of 0.80, 0.61 and 0.71, and reach an AUC of 0.76, 0.63 and 0.61 in EGFR E19/E21 prediction. The performance of optimal EGFR E19/E21 prediction model was not that satisfactory. We thought that data selection bias and limited sample size with relative high complexity and heterogeneity may lead to this result. Interestingly, we found that the models that performed best in the internal test group did not necessarily perform best in the two external test groups. Most of the existing studies were based on small samples or single-center data, which may lead to a locally optimal result. External validation *via* multi-center data could ensure the generalization performance of the model. Therefore, it also reflected that our optimal models had stable predictive performance.

Increasing evidence has verified that HRFs played a significant important role in predicting prognosis (34), EGFR mutation status and subtypes of NSCLC (13, 15, 17). Compared with Yang’s study (17), we extracted more complete HRFs and obtained better EGFR-related results. In terms of all the feature attributes, both our and Yang’s study found that wavelet features of CT imaging showed better predictive power. Also, some radiomics models were built using CT HRFs to identify E19 and E21 in lung adenocarcinoma (17, 35, 36), but their performance was difficult to compare due to the different medical modes and model evaluation indexes. Liu et al. (12) reported that 10 HRFs, which included seven wavelet features of CT imaging, two texture features of PET imaging, and one texture feature of CT imaging, were significantly associated with EGFR subtypes (E19 vs. E21). However, we partially selected the same feature categories but completely different features for prediction model building. In our research, we found that CT texture features based on wavelet filter had the biggest proportion in task I-II. As shown in previous studies, LBP features were popular descriptors in many images analysis tasks and showed pretty good contribution to model performance (37, 38). Wavelet features were also reported as a predictive marker in predicting treatment response and tumor phenotype of NSCLC (39, 40), which were in accordance with our work. LGBM and XGB classifiers always had better performance than LR models and RF models, which were accordance with previous studies (41).

The ability to interpret and validate automatic decisions-making of clinical predictive models is significant important in practical applications. Our results found that XAI can determine the most important features, further reveal the interactions of features that have an impact on model output, and then enhance clinical trustworthiness in predictive models. Yang et al. (42) established an explainable model to predict EGFR mutation status in lung adenocarcinoma based on seven machine learning algorithms and SHAP, but they only used semantic features, and did not involve LIME. Currently, many researches also have verified that SHAP and LIME were useful and effective approaches (25–27). Consistent with us, the results indicated that these two XAI approaches could pinpoint the most significant and important features of the model, and make the predictive model an effective and explainable tool for supporting clinical diagnosis and treatment.

Combined HRFs and clinical risk factors also have a satisfying predictive performance in prognosis. We also constructed a CPH model using 22 HRFs and 5 clinical risk factors to predict OS. Our model reached higher C-index than Yang’s (17) study (0.863 vs. 0.757). In our study, there is a significant difference in survival time between EGFR mutant and EGFR wild groups. This indicated that different EGFR mutation status and different treatment method may affect patients’ survival time. The survival analysis showed that the prognosis of targeted therapy was better than chemotherapy in patients with EGFR mutant type, which was consistent with previous studies (43, 44). However, there is no significant difference in survival time between patients with E19 and E21, furthermore, the survival rate of patients with E19 was better than that with E21, which was similar to previous study (45).

Several factors may explain the differences among the studies mentioned above. One reason may be the differences in imaging processing, VOIs segmentation in different studies. Most importantly, multi-center data has richer information and different PET/CT scanners and associated protocols, which increases the complexity and heterogeneity of data. On the one hand, it decreased the over fitting risk of model, on the other hand, it had an effect on the overall performance of model. This may be the major reason why the AUC performance of our optimal models was lower than that of some published studies (12–14).

Recent studies have shown that non-invasive approaches based on PET/CT, for example, analysis of ^18^F-FDG uptake (SUVmax, metabolic volume, tumor lesion glucose) and ^18^F-FDG PET/CT-derived radiomics (46, 47) could help quantify EGFR mutation status and predict prognosis in lung cancer patients for precision therapy. However, in some previous studies, the results of ^18^F-FDG uptake were conflicting (9, 10). The value of ^18^F-FDG uptake in EGFR mutation status prediction need to be evaluated in further high-quality studies. Radiomics extracted from diagnostic PET/CT images is a promising translational research field. Our study verified the feasibility and potential superiority of multi-center ^18^F-FDG PET/CT-derived radiomics to identify EGFR mutation status and predict prognosis in lung adenocarcinoma, which may help to develop a non-invasive tool as a complementary to PET/CT for clinic.

Our study has several limitations. First, although we collected data from 4 cohorts, it was a retrospective study and selection bias may have occurred. Unfortunately, we developed OS CPH model based on single-center data. In future work, it is necessary to enroll a larger multi-center/scanner data set with standard and complete follow-up information and perform a prospective analysis to evaluate our results, and further ensure better robustness and clinical applicability of the model. Second, the tumor regions were manually segmented slice by slice from radiologists, which could be time-consuming and susceptible to radiologist variability. There is an urgent need for an accurate semi-automatic segmentation method with unsupervised or weak supervision.

## Conclusions

In conclusion, we developed and interpreted two optimal predictive models to identify EGFR mutation status and subtypes in patients with lung adenocarcinoma based on cross-combination method and XAI technology, and further constructed a prognostic model to predict their clinical outcome.

## Data availability statement

The original contributions presented in the study are included in the article/**Supplementary Material**. Further inquiries can be directed to the corresponding author.

## Ethics statement

The study was performed in accordance with the ethical standards as laid down in the 1964 Declaration of Helsinki and its later amendments or comparable ethical standards. As our study is a retrospective analysis, informed consent is not required.

## Author contributions

All authors contributed to the study conception and design. Material preparation, data collection and analysis were performed by YZ and QL. The first draft of the manuscript was written by YZ and QL, and all authors commented on previous versions of the manuscript. All authors contributed to the article and approved the submitted version.

## Glossary

**Table:**

^18^F-FDG PET/CT	18 Fluorine-fluorodeoxyglucose positron emission tomography-computed tomography
EGFR	epidermal growth factor receptor
HRFs	handcrafted radiomics features
TKIs	tyrosine kinase inhibitors
E19	EGFR Exon 19 deletion
E21	EGFR exon 21 L858R missense
OS	overall survival
D	dimensions
XAI	explainable artificial intelligence
SHAP	Shapley additive explanations
LIME	local interpretable model-agnostic explanations
CPH	cox proportional hazard
VOIs	volumes of interest
LBP	local binary patterns
LoG	Laplacian of Gaussian
T	tumor
N	node
M	metastasis
PCC	Pearson correlation coefficient
RFE	recursive feature elimination
SFS	sequential forward selection
SBS	sequential backward selection
LGBM	light gradient boosting machine
XGB	extreme gradient boosting
KNN	K-nearest Neighbor
LASSO	Least absolute shrinkage and selection operator
SVM	support vector machine
RF	random forest
LR	logistic regression
TRIPOD	transparent reporting of a multivariable prediction model for individual prognosis or diagnosis
RQS	radiomics quality score
ROC	area under the receiver operating characteristic
AUC	area under the curve
DCA	decision curve analysis
LD	target lesion’s longest diameter
GLCM	gray-level co-occurrence matrix
GLRLM	gray-level run-length matrix
NGTDM	neighborhood gray tone difference matrix
GLSZM	gray-level size zone matrix
GLDM	gray-level dependence matrix.
