# Supplementary material for: Optimal 18F-FDG PET/CT radiomics model development for predicting EGFR mutation status and prognosis in lung adenocarcinoma: a multicentric study
Source: Front Oncol. 2023 May 8;13:1173355. doi: 10.3389/fonc.2023.1173355 (PMC10200887; doi:10.3389/fonc.2023.1173355)
Supplement: Supplementary file 1 [file DataSheet_1.docx]

**Supplemental files**

**Appendix** **E1. Inclusion and exclusion criteria**

The inclusion criteria were as follows: (i) histologically confirmed lung adenocarcinoma; (ii) pathologically confirmed EGFR mutation status; (iii) baseline PET and CT images obtained and complete clinical factors. The exclusion criteria included: (i) the tumor margin could not contour, especially in PET data; and (ii) they received lung cancer-related treatment, such as surgery, chemotherapy, radiotherapy, targeting, and immunotherapy.

## Appendix E2. Image acquisition

Before ^18^F-FDG administration, all patients underwent a glucose level test, and the blood glucose levels were less than 140 mg/dl. Then, the patients fasted for at least six hours before the injection of ^18^F-FDG, and image acquisition was started 1 hour afterward. During the scanning, the patient was in the head-first supine position, and the scanning range was from the skull base to 1/3 of the femur. After selecting the examination range, CT scanning was performed first, followed by PET scanning. The PET image datasets were reconstructed using CT data for attenuation correction.

## Appendix E3. Tumor segmentation and HRFs extraction

PET/CT images from public, SCH and RJ were segmented separately by two board-certified radiologists with 5 (reader 1) and 14 (reader 2) years of experience—a third chief physician (reader 3) reviewed. PET/CT images from FUSCC were segmented separately by another two board-certified radiologists with 5 (reader 4) and 12 (reader 5) years of experience—another third chief physician (reader 6) reviewed. The six radiologists were blinded to all examination results but referred to the diagnostic reports of the Department of Nuclear Medicine and annotation information of the public data set.

Both PET and CT HRFs were extracted from the original and 8 types of filter-based images, including wavelet, Laplacian of Gaussian (LoG), square, squareroot, local binary pattern (LBP) 3D, gradient, exponential and logarithm. In order to reduce the feature complexity, LBP 2D filter-based features were not extracted because of its lower accuracy than LBP 3D filter-based features [1]. To evaluate the robustness and repeatability of handcrafted radiomics features (HRFs) and avoid potential recall bias, random 10 cases from each of the 4 cohorts were selected, segmented again, and a re-segmentation set was constructed within one week. The intra –and interclass correlation coefficients values of HRFs were calculated.

**Appendix** **E4. Interpretation of optimal predictive models**

Feature importance, SHAP and LIME were used for model interpretation. Feature importance can be explained through the weights and can be done on overall features. The rank of feature importance was strongly associated with the predictive model. SHAP could evaluate the positive and negative influence of features on the model output by calculating Shapley value. The larger the Shapley value, the higher the rank of feature importance would be. The higher the value of the feature, the redder the color of the sample point. LIME constructed a linear surrogate model using the sample and its nearest neighbors, which fit the local boundary of the complex predictive model that need interpretation.

In this study, feature_importance function of Python Scikit-learn framework was used to calculate optimal models’ feature importance. To interpret tree ensemble models (LGBM and XGB), we adopt TreeSHAP with feature_perturbation = “tree_path_dependent” (SHAP version 0.41.0). Hence, the positive and negative influence of each feature on the model output can be obtained. To locally explain optimal predictive models, lime_tabular.LIMETabularExplainer function (LIME version 0.2.0.1) was employed.

**References**

1. Kohei A, Yeni H, Hiroshi O. Comparison of 2D and 3D Local Binary Pattern in Lung Cancer Diagnosis. International Journal of Advanced Computer Science and Applications. 2012;3(4).


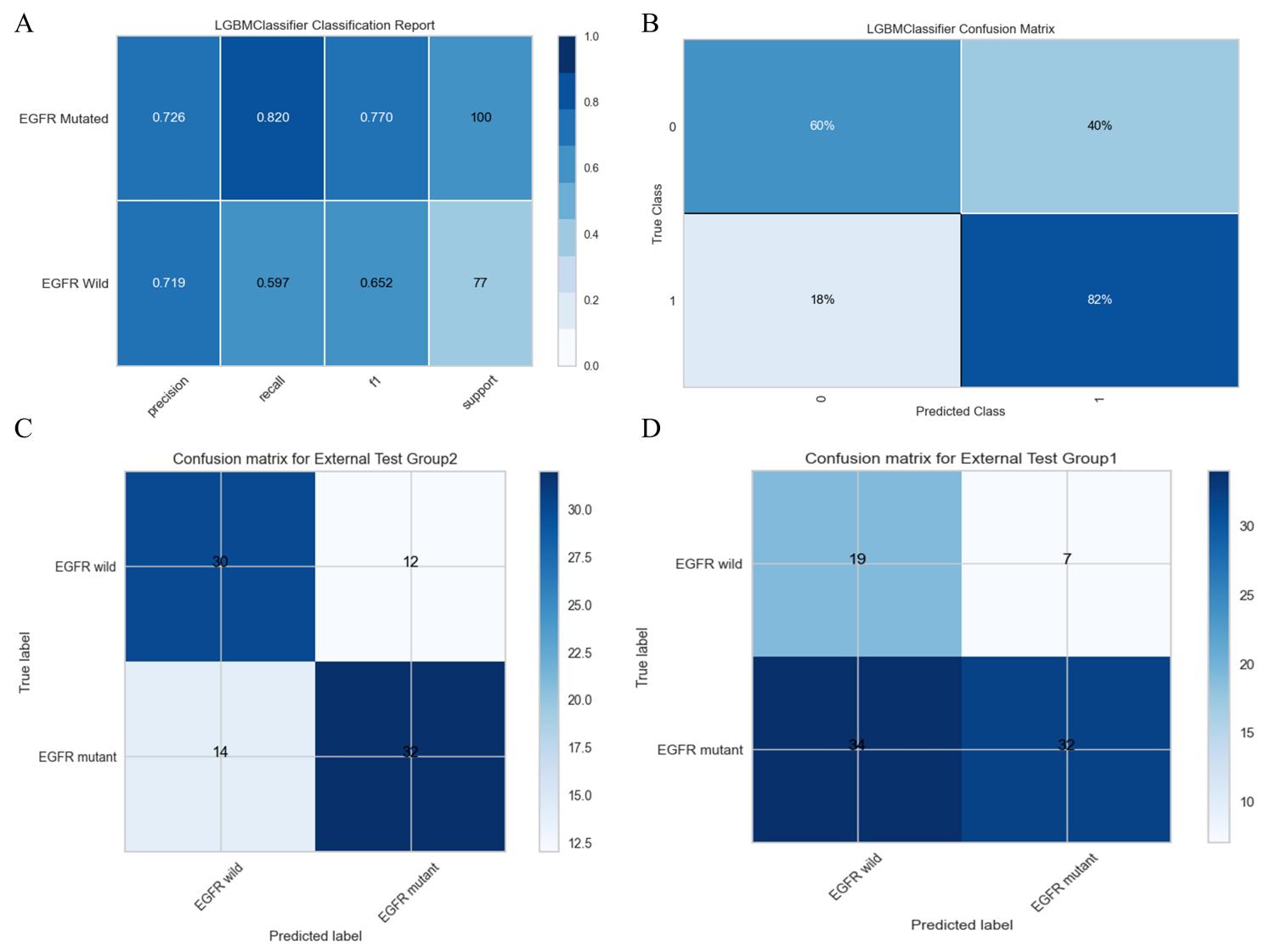
Fig. S1 Classification report and confusion matrix of optimal model in predicting EGFR mutation status.

A, Classification report of internal test group of optimal predictive model. B-D, Confusion matrix of internal test group, external test group 1 and external test group 2.

Fig. S2 The correlation heatmap of 29 HRFs of optimal model in task Ⅰ.


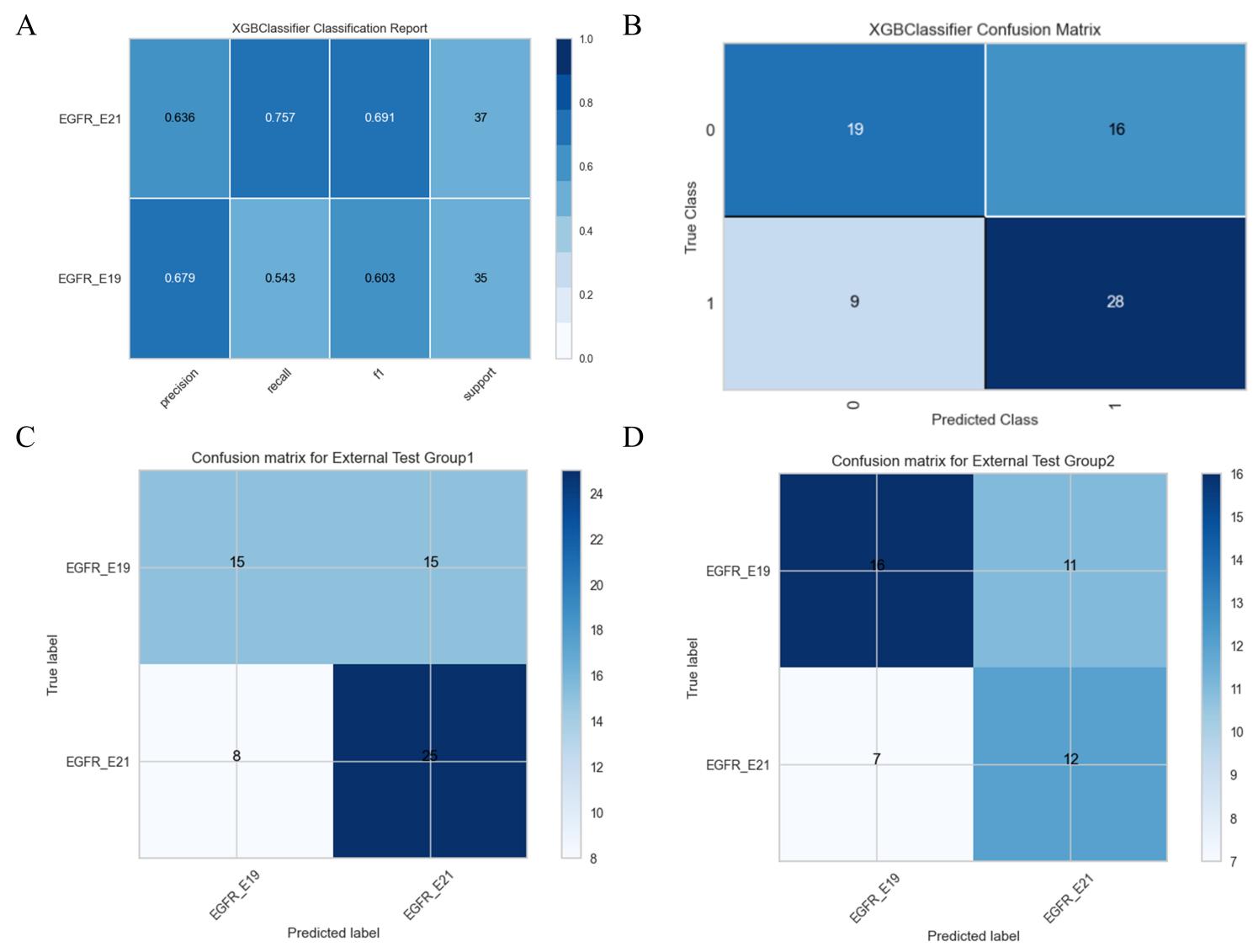


Fig. S3 Classification report and confusion matrix of optimal model in predicting EGFR mutation subtypes.

A, Classification report of internal test group of optimal predictive model. B-D, Confusion matrix of internal test group, external test group 1 and external test group 2.

**
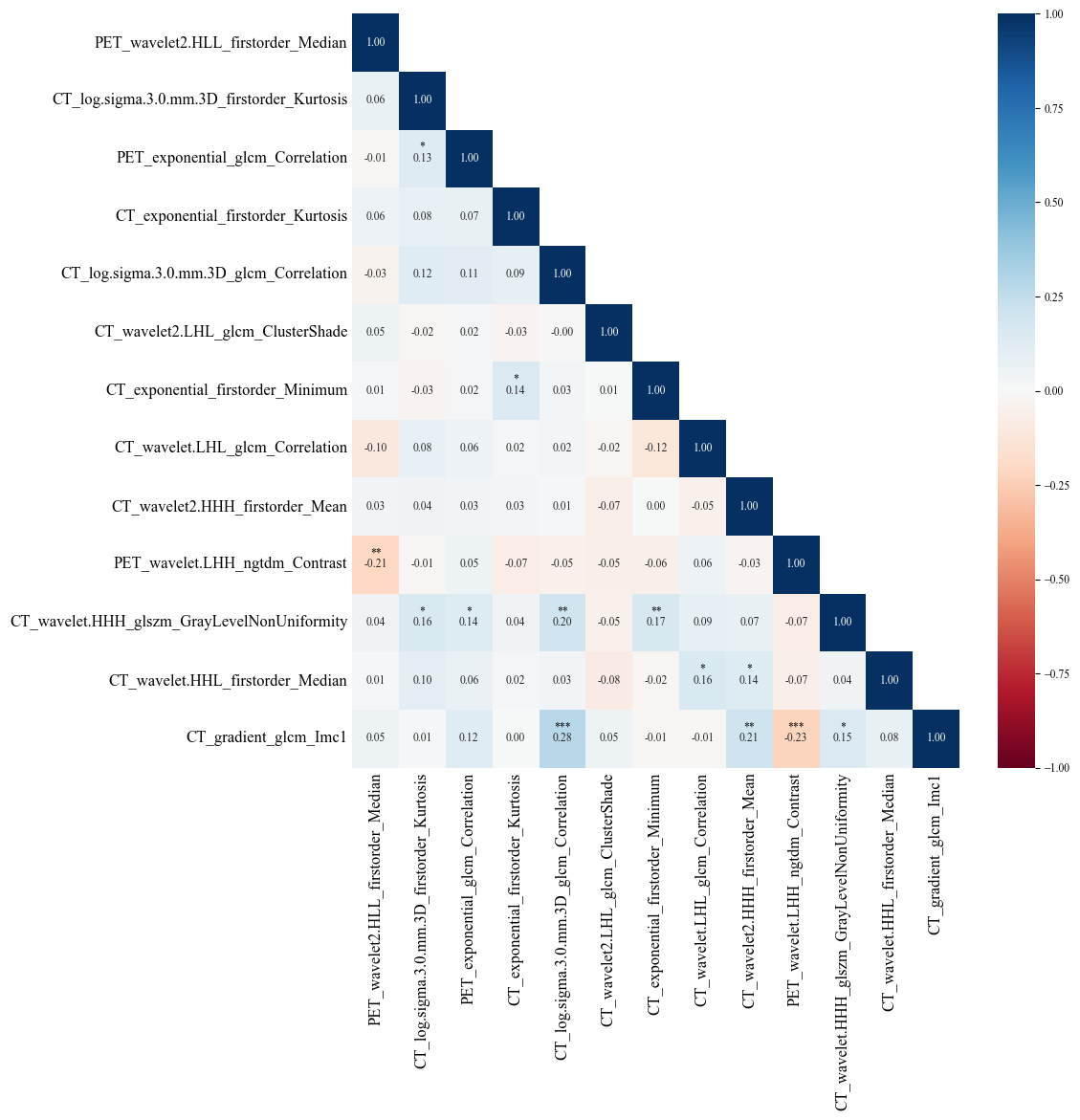
**

Fig. S4 The correlation heatmap of 13 HRFs of optimal model in task Ⅱ.

Fig. S5 The coefficients of Log Lambda of LASSO feature selection in task Ⅲ.

Fig. S6 The Hazard ratio of multicox HRFs in task Ⅲ.

## Table S1 Scanner information and imaging protocols

| Characteristics | SCH | FUSCC | RJ | Public |
| --- | --- | --- | --- | --- |
| Equipment | SIEMENS  Biograph mCT | SIEMENS  Biogragh 16HR | SIEMENS  Biograph mCT-s | SIEMENS/GE/Philips  Medical Systems |
| Reconstruction | PSF | OSEM  (Iterations 4; subsets 8) | PSF+TOF | * |
| Tube Voltage (CT) | 120 kV | 120 kV | 120 kV | * |
| Tube Current (CT) | 140 mA | 140 mA | Adjusted by CAREDose 4D technology | * |
| Slice thickness (PET, mm) | 5.00 | 5.00 | 5.00 | 3.27-5.00 |
| Slice thickness (CT, mm) | 3.00 | 5.00 | 3.00 | 3.27-5.00 |
| Image size (PET, pixel) | 200*200 | 168*168/192*192/200*200 | 200*200 | 128*128 / 144*144  168*168 / 192*192 |
| Image size (CT, pixel) | 512*512 | 512*512 | 512*512 | 512*512 |
| Acquisition time | 3 min/bed position | 2–3 min/bed position | 2 minutes/bed position | * |
| Convolution kernel | Gauss 2.00 | Gauss 5.00 | Gauss 4.00 | * |

Annotations: PSF, point spread function; OSEM, ordered subset expectation maximization; TOF, time of flight; mm, millimeter; GE, General Electric Company.

## Table S2 Parameters of params.yaml of CT HRFs and PET HRFs

| Parameters | CT | PET |
| --- | --- | --- |
| imageType | Original  Wavelet: {start_level: 1; level: 2; wavelet: coif3}  LBP 3D  LoG  Square  SquareRoot  Gradient  Exponential  Logarithm | Original  Wavelet: {start_level: 1; level: 2; wavelet: "coif3"}  LBP 3D  LoG  Square  SquareRoot  Gradient  Exponential  Logarithm |
| featureClass | shape  firstorder  glcm  glrlm  ngtdm  glszm  gldm | shape  firstorder  glcm  glrlm  ngtdm  glszm  gldm |
| binWidth | 25 | 128 |
| interpolator | 'sitkBSpline' | 'sitkBSpline' |
| resampledPixelSpacing | [1, 1, 1] | [1, 1, 1] |
| geometryTolerance | 0.1 | 0.1 |
| voxelArrayShift | 1000 | 1000 |
| label | 1 | 1 |

## Table S3 List of Extracted Handcrafted Radiomics Features.

| **Classification (Total number=2380)** | **Features** |
| --- | --- |
| **First Order Features (18)** | Energy TotalEnergy Entropy Minimum 10thpercentile 90thpercentile Maximum Mean Median Interquartile_Range Range Skewness Kurtosis Variance Uniformity  Mean Absolute Deviation (MAD)  Robust Mean Absolute Deviation (rMAD) Root Mean Squared (RMS) |
| **Shape Features (14)** | Elongation Flatness LeastAxisLength MajorAxisLength  Maximum2DDiameterColumn Maximum2DDiameterRow VoxelVolume  Maximum2DDiameterSlice Maximum3DDiameter shape_MeshVolume  MinorAxisLength Sphericity SurfaceArea SurfaceVolumeRatio |
| **Textural Features (22+16+16+14+5=73)** | **22 features from GLCM:**  Autocorrelation Joint Average Cluster Prominence Cluster Shade  Cluster Tendency Contrast Correlation Difference Average  Difference Entropy Difference Variance Joint Energy Joint Entropy  Informational Measure of Correlation (IMC) 1  Informational Measure of Correlation (IMC) 2  Inverse Difference Moment (IDM)  Maximal Correlation Coefficient (MCC)  Inverse Difference Moment Normalized (IDMN)  Inverse Difference (ID) Inverse Variance (IV)  Maximum Probability Sum Entropy Sum of Squares  **16 features from GLRLM:**  Short Run Emphasis (SRE)  Long Run Emphasis (LRE)  Gray Level Non-Uniformity (GLN)  Gray Level Non-Uniformity Normalized (GLNN)  Run Length Non-Uniformity (RLN)  Run Length Non-Uniformity Normalized (RLNN)  Run Percentage (RP)  Gray Level Variance (GLV)  Run Variance (RV)  Run Entropy (RE)  Low Gray Level Run Emphasis (LGLRE)  High Gray Level Run Emphasis (HGLRE)  Short Run Low Gray Level Emphasis (SRLGLE)  Short Run High Gray Level Emphasis (SRHGLE)  Long Run Low Gray Level Emphasis (LRLGLE)  Long Run High Gray Level Emphasis (LRHGLE)  **16 features from GLSZM:**  Small Area Emphasis (SAE)  Large Area Emphasis (LAE)  Gray Level Non-Uniformity (GLN)  Gray Level Non-Uniformity Normalized (GLNN)  Size-Zone Non-Uniformity (SZN)  Size-Zone Non-Uniformity Normalized (SZNN)  Zone Percentage (ZP)  Gray Level Variance (GLV)  Zone Variance (ZV)  Zone Entropy (ZE)  Low Gray Level Zone Emphasis (LGLZE)  High Gray Level Zone Emphasis (HGLZE)  Small Area Low Gray Level Emphasis (SALGLE)  Small Area High Gray Level Emphasis (SAHGLE)  Large Area Low Gray Level Emphasis (LALGLE)  Large Area High Gray Level Emphasis (LAHGLE)  **14 features from GLDM:**  Small Dependence Emphasis (SDE)  Large Dependence Emphasis (LDE)  Gray Level Non-Uniformity (GLN)  Dependence Non-Uniformity (DN)  Dependence Non-Uniformity Normalized (DNN)  Gray Level Variance (GLV)  Dependence Variance (DV)  Dependence Entropy (DE)  Low Gray Level Emphasis (LGLE)  High Gray Level Emphasis (HGLE)  Small Dependence Low Gray Level Emphasis (SDLGLE)  Small Dependence High Gray Level Emphasis (SDHGLE)  Large Dependence Low Gray Level Emphasis (LDLGLE)  Large Dependence High Gray Level Emphasis (LDHGLE)  **5 features from NGTDM:**  Coarseness Contrast Busyness Complexity Strength |
| **Wavelet features (91*15=1365)** | **15 decompositions:**  For each decomposition, all of the above features except shape features were computed. |
| **LoG features (91*2)** | **2 decompositions:**  For each decomposition, all of the above features except shape features were computed. |
| **Square features (91)** | **1 decomposition:**  For each decomposition, all of the above features except shape features were computed. |
| **SquareRoot features (91)** | **1 decomposition:**  For each decomposition, all of the above features except shape features were computed. |
| **Local Binary Pattern (3D) features (91*3=273)** | **3 decompositions:**  For each decomposition, all of the above features except shape features were computed. |
| **Gradient features (91)** | **1 decomposition:**  For each decomposition, all of the above features except shape features were computed. |
| **Exponential features (91)** | **1 decomposition:**  For each decomposition, all of the above features except shape features were computed. |
| **Logarithm features (91)** | **1 decomposition:**  For each decomposition, all of the above features except shape features were computed. |

**Table S4. Performance of feature selection methods in Task Ⅰ and Task Ⅱ**

| FS methods | Task Ⅰ | |  | Task Ⅱ | |
| --- | --- | --- | --- | --- | --- |
|  | Count | AUC Threshold |  | Count | AUC Threshold |
| XGB | 17 | 0.6812 |  | 20 | 0.5913 |
| RF | 17 | 0.6723 |  | 30 | 0.5758 |
| LGBM | 18 | 0.6707 |  | 20 | 0.5844 |
| LR | 22 | 0.6041 |  | 15 | 0.5712 |
| SVM | 21 | 0.6510 |  | 13 | 0.5787 |
| KNN | 24 | 0.6601 |  | 20 | 0.5624 |
| RFE-XGB | 20 | 0.6997 |  | 27 | 0.5783 |
| RFE-RF | 18 | 0.6918 |  | 14 | 0.5791 |
| RFE-LGBM | 29 | 0.7393 |  | 18 | 0.6227 |
| RFE-LR | 15 | 0.5700 |  | 26 | 0.5822 |
| SFS-XGB | 21 | 0.8147 |  | 8 | 0.8075 |
| SFS-RF | 20 | 0.8432 |  | 19 | 0.8353 |
| SFS-LGBM | 28 | 0.8228 |  | 25 | 0.8669 |
| SFS-LR | 13 | 0.7402 |  | 13 | 0.6931 |
| SBS-XGB | 18 | 0.8517 |  | 27 | 0.8770 |
| SBS-RF | 11 | 0.8164 |  | 15 | 0.8259 |
| SBS-LGBM | 24 | 0.8561 |  | 23 | 0.9161 |
| SBS-LR | 30 | 0.8000 |  | 29 | 0.8909 |
| LASSO | 20 | - |  | 20 | - |

Abbreviation: FI, feature importance. AVG, average.

**Table S5. Interquartile range of the enrolled radiomic features of the optimal model in task Ⅰ**

| Number | Enrolled HRFs | Train | Internal Test | External Test1 | External Test2 |
| --- | --- | --- | --- | --- | --- |
| F1 | CT_exponential_firstorder_Minimum | [-0.16--0.087] | [-0.164--0.094] | [-0.441--0.045] | [-0.188--0.054] |
| F2 | CT_gradient_glszm_GrayLevelNonUniformityNormalized | [-0.363-0.095] | [-0.331-0.075] | [-0.378--0.003] | [-0.701-0.423] |
| F3 | CT_lbp.3D.k_glcm_Correlation | [-0.433-0.261] | [-0.384-0.31] | [-0.348-0.486] | [-0.66--0.111] |
| F4 | CT_lbp.3D.k_glrlm_LongRunEmphasis | [-0.394-0.085] | [-0.367-0.126] | [-0.839-0.792] | [-0.616-0.186] |
| F5 | CT_log.sigma.1.0.mm.3D_gldm_LowGrayLevelEmphasis | [-0.59-0.313] | [-0.599-0.16] | [-0.658-0.473] | [-0.709-0.401] |
| F6 | CT_logarithm_firstorder_10Percentile | [-0.395-0.254] | [-0.352-0.247] | [-0.226-0.518] | [-0.467-0.055] |
| F7 | CT_logarithm_ngtdm_Strength | [-0.616-0.314] | [-0.613-0.443] | [-0.539--0.04] | [-0.845-0.707] |
| F8 | CT_wavelet.HHH_glszm_SmallAreaEmphasis | [-1.14-0.713] | [-0.951-0.855] | [-0.525-0.693] | [-0.644-0.85] |
| F9 | CT_wavelet.HLL_firstorder_Kurtosis | [-0.383--0.005] | [-0.399-0.003] | [-0.558-0.116] | [-0.683-0.527] |
| F10 | CT_wavelet.LLH_gldm_LargeDependenceHighGrayLevelEmphasis | [-0.33--0.114] | [-0.331--0.062] | [-0.652-0.379] | [-0.593-0.05] |
| F11 | CT_wavelet2.HHH_glszm_SizeZoneNonUniformityNormalized | [-0.764-0.614] | [-0.764-0.619] | [-0.622-0.618] | [-0.95-0.824] |
| F12 | CT_wavelet2.LHH_firstorder_RootMeanSquared | [-0.519-0.276] | [-0.439-0.31] | [-0.608-0.34] | [-0.425-0.174] |
| F13 | CT_wavelet2.LHH_glcm_ClusterShade | [-0.095-0.146] | [-0.057-0.201] | [-0.238-0.081] | [-0.054-0.014] |
| F14 | CT_wavelet2.LLL_glcm_ClusterShade | [-0.338-0.533] | [-0.491-0.479] | [-0.805-0.69] | [-0.261-0.421] |
| F15 | PET_exponential_firstorder_10Percentile | [-0.332-0.01] | [-0.328-0.031] | [-0.506--0.056] | [-0.523-0.052] |
| F16 | PET_exponential_firstorder_90Percentile | [-0.635-0.231] | [-0.636-0.263] | [-0.723-0.254] | [-0.602-0.212] |
| F17 | PET_lbp.3D.k_firstorder_Kurtosis | [-0.611-0.412] | [-0.55-0.27] | [-0.833-0.56] | [-0.64-0.342] |
| F18 | PET_original_firstorder_10Percentile | [-0.661-0.505] | [-0.798-0.33] | [-0.873-0.953] | [-0.657-0.42] |
| F19 | PET_square_gldm_DependenceVariance | [-0.394--0.021] | [-0.373-0.017] | [-0.382--0.055] | [-0.491-0.117] |
| F20 | PET_wavelet.HLH_firstorder_Median | [-0.345-0.29] | [-0.319-0.193] | [-0.343-0.176] | [-0.208-0.398] |
| F21 | PET_wavelet.HLH_glcm_ClusterShade | [-0.061-0.044] | [-0.027-0.041] | [-0.036--0.023] | [-0.011-0.041] |
| F22 | PET_wavelet.HLL_firstorder_Median | [-0.47-0.355] | [-0.25-0.508] | [-0.445-0.283] | [-0.449-0.356] |
| F23 | PET_wavelet.LHH_firstorder_Mean | [-0.244-0.308] | [-0.107-0.383] | [-0.215-0.21] | [-0.454-0.307] |
| F24 | PET_wavelet.LHL_gldm_LargeDependenceLowGrayLevelEmphasis | [-0.293--0.166] | [-0.293--0.118] | [-0.334--0.158] | [-0.296--0.204] |
| F25 | PET_wavelet2.HLH_firstorder_Kurtosis | [-0.608-0.28] | [-0.578-0.197] | [-0.757-0.485] | [-0.609-0.357] |
| F26 | PET_wavelet2.HLL_glcm_Correlation | [-0.55-0.581] | [-0.42-0.648] | [-0.505-0.629] | [-0.651-0.686] |
| F27 | PET_wavelet2.LHH_firstorder_Kurtosis | [-0.524-0.137] | [-0.531-0.247] | [-0.762-0.468] | [-0.884-0.89] |
| F28 | PET_wavelet2.LHH_glszm_SmallAreaLowGrayLevelEmphasis | [-0.681-0.332] | [-0.713-0.304] | [-0.819-0.529] | [-0.758-0.404] |
| F29 | PET_wavelet2.LLH_glcm_ClusterShade | [0.07-0.104] | [0.07-0.086] | [0.099-0.101] | [0.085-0.089] |

**Table S6.** **Interquartile range of the enrolled radiomic features of the optimal model in task Ⅱ**

| Number | Enrolled HRFs | Train | Internal Test | External Test1 | External Test2 |
| --- | --- | --- | --- | --- | --- |
| F1 | PET_wavelet2.HLL_firstorder_Median | [-0.209--0.201] | [-0.212--0.201] | [-0.143--0.142] | [-0.217--0.1] |
| F2 | CT_log.sigma.3.0.mm.3D_firstorder_Kurtosis | [-0.172--0.072] | [-0.174--0.076] | [-0.388--0.076] | [-0.783-0.397] |
| F3 | PET_exponential_glcm_Correlation | [-0.227-0.67] | [-0.849-0.626] | [-0.148-0.583] | [-0.64-0.898] |
| F4 | CT_exponential_firstorder_Kurtosis | [-0.713-0.546] | [-0.654-0.456] | [-0.516-0.091] | [-0.71-0.393] |
| F5 | CT_log.sigma.3.0.mm.3D_glcm_Correlation | [-0.183-0.511] | [-0.63-0.467] | [-0.619-0.76] | [-0.891-0.834] |
| F6 | CT_wavelet2.LHL_glcm_ClusterShade | [-0.592-0.518] | [-0.592-0.326] | [-0.152--0.149] | [-0.47-0.005] |
| F7 | CT_exponential_firstorder_Minimum | [-0.331-0.324] | [-0.126-0.602] | [-0.256-0.345] | [-0.117-0.422] |
| F8 | CT_wavelet.LHL_glcm_Correlation | [-0.604-0.549] | [-0.501-0.49] | [-0.648-0.552] | [-0.398-0.74] |
| F9 | CT_wavelet2.HHH_firstorder_Mean | [-0.343-0.267] | [-0.224-0.409] | [-0.265-0.192] | [-0.425--0.03] |
| F10 | PET_wavelet.LHH_ngtdm_Contrast | [-0.132-0.133] | [-0.236-0.107] | [-0.173-0.261] | [-0.145-0.346] |
| F11 | CT_wavelet.HHH_glszm_GrayLevelNonUniformity | [-0.669-0.432] | [-0.72-0.815] | [-0.762-0.566] | [-0.45-0.335] |
| F12 | CT_wavelet.HHL_firstorder_Median | [-0.492-0.086] | [-0.495-0.221] | [-0.64-0.308] | [-0.478--0.129] |
| F13 | CT_gradient_glcm_Imc1 | [-0.427-0.561] | [-0.711-0.3] | [-0.194-0.381] | [-0.363-0.457] |

**Table S7. Description of the enrolled radiomic features of the optimal model in task Ⅲ**

| **Number** | **Factors** | **mean** | **std** | **min** | **25%** | **75%** | **max** | **Coef** |
| --- | --- | --- | --- | --- | --- | --- | --- | --- |
| F1 | CT_exponential_firstorder_Kurtosis | 0 | 1 | -0.175 | -0.174 | -0.168 | 16.434 | 0.061 |
| F2 | CT_gradient_firstorder_10Percentile | 0 | 1 | -0.965 | -0.802 | 0.593 | 5.081 | -0.098 |
| F3 | CT_lbp.3D.k_firstorder_90Percentile | 0 | 1 | -2.324 | -0.783 | 0.785 | 3.762 | 0.055 |
| F4 | CT_square_glszm_SmallAreaLowGrayLevelEmphasis | 0 | 1 | -1.88 | -0.569 | 0.382 | 9.815 | 0.028 |
| F5 | CT_wavelet.HHH_glszm_SmallAreaEmphasis | 0 | 1 | -1.278 | -1.12 | 0.854 | 2.459 | 0.011 |
| F6 | CT_wavelet.HHL_firstorder_Skewness | 0 | 1 | -11.599 | -0.166 | 0.247 | 3.277 | 0.001 |
| F7 | CT_wavelet.LHL_firstorder_Skewness | 0 | 1 | -9.342 | -0.352 | 0.349 | 6.672 | 0.010 |
| F8 | CT_wavelet2.LLL_firstorder_90Percentile | 0 | 1 | -2.914 | -0.657 | 0.783 | 2.245 | 0.079 |
| F9 | PET_exponential_glszm_LargeAreaEmphasis | 0 | 1 | -0.122 | -0.119 | -0.089 | 18.884 | 0.012 |
| F10 | PET_exponential_glszm_ZoneVariance | 0 | 1 | -0.209 | -0.201 | -0.105 | 13.758 | 0.044 |
| F11 | PET_gradient_firstorder_Skewness | 0 | 1 | -3.361 | -0.609 | 0.585 | 3.661 | 0.032 |
| F12 | PET_gradient_glcm_Correlation | 0 | 1 | -3.083 | -0.709 | 0.759 | 2.194 | 0.139 |
| F13 | PET_original_shape_Sphericity | 0 | 1 | -3.936 | -0.523 | 0.734 | 1.68 | -0.211 |
| F14 | PET_square_gldm_DependenceVariance | 0 | 1 | -0.56 | -0.393 | -0.008 | 12.148 | 0.170 |
| F15 | PET_wavelet.HHH_glcm_Idmn | 0 | 1 | -1.921 | -1.14 | 0.889 | 1.729 | 0.002 |
| F16 | PET_wavelet.LHH_firstorder_Kurtosis | 0 | 1 | -1.188 | -0.702 | 0.417 | 5.338 | 0.017 |
| F17 | PET_wavelet.LHH_glszm_SizeZoneNonUniformityNormalized | 0 | 1 | -1.902 | -0.698 | 0.671 | 5.29 | 0.011 |
| F18 | PET_wavelet.LLH_firstorder_10Percentile | 0 | 1 | -7.549 | -0.164 | 0.556 | 1.624 | -0.112 |
| F19 | PET_wavelet2.HHH_glcm_Idmn | 0 | 1 | -2.009 | -1.189 | 0.833 | 1.937 | 0.009 |
| F20 | PET_wavelet2.HHL_ngtdm_Busyness | 0 | 1 | -0.573 | -0.439 | 0 | 9.586 | -0.001 |
| F21 | PET_wavelet2.HLH_glszm_SizeZoneNonUniformityNormalized | 0 | 1 | -1.838 | -0.679 | 0.665 | 4.387 | 0.057 |
| F22 | PET_wavelet2.LHL_firstorder_Kurtosis | 0 | 1 | -0.653 | -0.465 | 0.035 | 9.618 | 0.178 |
| PETCTRad_score | | 3.003 | 11.078 | 0.009 | 0.392 | 2.565 | 202.715 | - |
| RiskScore | | 1.367 | 1.682 | 0.25 | 0.583 | 1.604 | 24.789 | - |

**Table S8. The criteria and maximal radiomic quality score as well as the actural score of this work**

| **Criteria** | **Points system** | **Maximal score** | **Actual score of this work** |
| --- | --- | --- | --- |
| Image protocol quality - well-documented image protocols (for example, contrast, slice thickness, energy, etc.) and/or usage of public image protocols allow reproducibility/replicability | + 1 (if protocols are well-documented) + 1 (if public protocol is used) | 2 | 2 |
| Multiple segmentations - possible actions are: segmentation by different physicians/algorithms /software, perturbing segmentations by (random) noise, segmentation at different breathing cycles. Analyse feature robustness to segmentation variabilities | 1 | 1 | 1 |
| Phantom study on all scanners - detect inter-scanner differences and vendor-dependent features. Analyse feature robustness to these sources of variability | 1 | 1 | 0 |
| Imaging at multiple time points - collect images of individuals at additional time points. Analyse feature robustness to temporal variabilities (for example, organ movement, organ expansion /shrinkage) | 1 | 1 | 0 |
| Feature reduction or adjustment for multiple testing - decreases the risk of overfitting. Overfitting is inevitable if the number of features exceeds the number of samples. Consider feature robustness when selecting features | − 3 (if neither measure is implemented) + 3 (if either measure is implemented) | 3 | 3 |
| Multivariable analysis with non radiomics features (for example, EGFR mutation) - is expected to provide a more holistic model. Permits correlating /inferencing between radiomics and non radiomics features | 1 | 1 | 1 |
| Detect and discuss biological correlates - demonstration of phenotypic differences (possibly associated with underlying gene–protein expression patterns) deepens understanding of radiomics and biology | 1 | 1 | 1 |
| Cut-off analyses - determine risk groups by either the median, a previously published cut-off or report a continuous risk variable. Reduces the risk of reporting overly optimistic results | 1 | 1 | 1 |
| Discrimination statistics - report discrimination statistics (for example, C-statistic, ROC curve, AUC) and their statistical significance (for example, p-values, confidence intervals). One can also apply resampling method (for example, bootstrapping, cross-validation) | + 1 (if a discrimination statistic and its statistical significance are reported) + 1 (if a resampling method technique is also applied) | 2 | 2 |
| Calibration statistics - report calibration statistics (for example, Calibration-in-the-large/slope, calibration plots) and their statistical significance (for example, P-values, confidence intervals). One can also apply resampling method (for example, bootstrapping, cross-validation) | + 1 (if a calibration statistic and its statistical significance are reported) + 1 (if a resampling method technique is also applied) | 2 | 2 |
| Prospective study registered in a trial database - provides the highest level of evidence supporting the clinical validity and usefulness of the radiomics biomarker | + 7 (for prospective validation of a radiomics signature in an appropriate trial) | 7 | 0 |
| Validation - the validation is performed without retraining and without adaptation of the cut-off value, provides crucial information with regard to credible clinical performance | - 5 (if validation is missing) + 2 (if validation is based on a dataset from the same institute) + 3 (if validation is based on a dataset from another institute) + 4 (if validation is based on two datasets from two distinct institutes) + 4 (if the study validates a previously published signature) + 5 (if validation is based on three or more datasets from distinct institutes) | 5 | 4 |
| Comparison to 'gold standard' - assess the extent to which the model agrees with/is superior to the current 'gold standard' method (for example, TNM-staging for survival prediction). This comparison shows the added value of radiomics | 2 | 2 | 2 |
| Potential clinical utility - report on the current and potential application of the model in a clinical setting (for example, decision curve analysis) | 2 | 2 | 2 |
| Cost-effectiveness analysis - report on the cost-effectiveness of the clinical application (for example, QALYs generated) | 1 | 1 | 0 |
| Open science and data - make code and data publicly available. Open science facilitates knowledge transfer and reproducibility of the study | + 1 (if scans are open source) +1 (if region of interest segmentations are open source) + 1 (if code is open source) + 1 (if radiomics features are calculated on a set of representative ROIs and the calculated features and representative ROIs are open source) | 4 | 2 |
| Total score |  | 36 | 23 |

**Table S9. TRIPOD Checklist: Prediction Model Development and Validation**

| **Section/Topic** | **Item** |  | **Checklist Item** | **Page** |
| --- | --- | --- | --- | --- |
| **Title and abstract** | | | | |
| Title | 1 | D; V | Identify the study as developing and/or validating a multivariable prediction model, the target population, and the outcome to be predicted. | Yes |
| Abstract | 2 | D; V | Provide a summary of objectives, study design, setting, participants, sample size, predictors, outcome, statistical analysis, results, and conclusions. | Yes |
| **Introduction** | | | | |
| Background and objectives | 3a | D; V | Explain the medical context (including whether diagnostic or prognostic) and rationale for developing or validating the multivariable prediction model, including references to existing models. | Yes |
|  | 3b | D; V | Specify the objectives, including whether the study describes the development or validation of the model or both. | Yes |
| **Methods** | | | | |
| Source of data | 4a | D; V | Describe the study design or source of data (e.g., randomized trial, cohort, or registry data), separately for the development and validation data sets, if applicable. | Yes |
|  | 4b | D; V | Specify the key study dates, including start of accrual; end of accrual; and, if applicable, end of follow-up. | Yes |
| Participants | 5a | D; V | Specify key elements of the study setting (e.g., primary care, secondary care, general population) including number and location of centres. | Yes |
|  | 5b | D; V | Describe eligibility criteria for participants. | Yes |
|  | 5c | D; V | Give details of treatments received, if relevant. | Yes |
| Outcome | 6a | D; V | Clearly define the outcome that is predicted by the prediction model, including how and when assessed. | Yes |
|  | 6b | D; V | Report any actions to blind assessment of the outcome to be predicted. | NaN |
| Predictors | 7a | D; V | Clearly define all predictors used in developing or validating the multivariable prediction model, including how and when they were measured. | Yes |
|  | 7b | D; V | Report any actions to blind assessment of predictors for the outcome and other predictors. | NaN |
| Sample size | 8 | D; V | Explain how the study size was arrived at. | Yes |
| Missing data | 9 | D; V | Describe how missing data were handled (e.g., complete-case analysis, single imputation, multiple imputation) with details of any imputation method. | Yes |
| Statistical analysis methods | 10a | D | Describe how predictors were handled in the analyses. | Yes |
|  | 10b | D | Specify type of model, all model-building procedures (including any predictor selection), and method for internal validation. | Yes |
|  | 10c | V | For validation, describe how the predictions were calculated. | Yes |
|  | 10d | D; V | Specify all measures used to assess model performance and, if relevant, to compare multiple models. | Yes |
|  | 10e | V | Describe any model updating (e.g., recalibration) arising from the validation, if done. | NaN |
| Risk groups | 11 | D; V | Provide details on how risk groups were created, if done. | NaN |
| Development vs. validation | 12 | V | For validation, identify any differences from the development data in setting, eligibility criteria, outcome, and predictors. | Yes |
| **Results** | | | | |
| Participants | 13a | D; V | Describe the flow of participants through the study, including the number of participants with and without the outcome and, if applicable, a summary of the follow-up time. A diagram may be helpful. | Yes |
|  | 13b | D; V | Describe the characteristics of the participants (basic demographics, clinical features, available predictors), including the number of participants with missing data for predictors and outcome. | Yes |
|  | 13c | V | For validation, show a comparison with the development data of the distribution of important variables (demographics, predictors and outcome). | Yes |
| Model development | 14a | D | Specify the number of participants and outcome events in each analysis. | Yes |
|  | 14b | D | If done, report the unadjusted association between each candidate predictor and outcome. | Yes |
| Model specification | 15a | D | Present the full prediction model to allow predictions for individuals (i.e., all regression coefficients, and model intercept or baseline survival at a given time point). | Yes |
|  | 15b | D | Explain how to the use the prediction model. | Yes |
| Model performance | 16 | D; V | Report performance measures (with CIs) for the prediction model. | Yes |
| Model-updating | 17 | V | If done, report the results from any model updating (i.e., model specification, model performance). | NaN |
| **Discussion** | | | | |
| Limitations | 18 | D; V | Discuss any limitations of the study (such as nonrepresentative sample, few events per predictor, missing data). | Yes |
| Interpretation | 19a | V | For validation, discuss the results with reference to performance in the development data, and any other validation data. | Yes |
|  | 19b | D; V | Give an overall interpretation of the results, considering objectives, limitations, results from similar studies, and other relevant evidence. | Yes |
| Implications | 20 | D; V | Discuss the potential clinical use of the model and implications for future research. | Yes |
| **Other information** | | | | |
| Supplementary information | 21 | D; V | Provide information about the availability of supplementary resources, such as study protocol, Web calculator, and data sets. | Yes |
| Funding | 22 | D; V | Give the source of funding and the role of the funders for the present study. | Yes |
